# Mapping the Non‐Canonical Splicing Variants: Decrypting the Hidden Genetic Architecture of Idiopathic Male Infertility

**DOI:** 10.1002/advs.202515512

**Published:** 2025-10-30

**Authors:** Kuokuo Li, Yuge Chen, Dongdong Tang, Yuying Sheng, Xu Han, Hao Geng, Na Zhang, Zongliu Duan, Guanxiong Wang, Yang Gao, Rui Guo, Rong Hua, Zhiming Ding, Chuan Xu, Qunshan Shen, Zhen Yu, Bing Song, Mingrong Lv, Yuping Xu, Huan Wu, Ji Wu, Yunxia Cao, Xiaojin He

**Affiliations:** ^1^ Reproductive Medicine Center Department of Obstetrics and Gynecology The First Affiliated Hospital of Anhui Medical University No. 218 Jixi Road Hefei Anhui 230022 China; ^2^ NHC Key Laboratory of Study on Abnormal Gametes and Reproductive Tract (Anhui Medical University) No. 81 Meishan Road Hefei Anhui 230022 China; ^3^ Engineering Research Center of Biopreservation and Artificial Organs Ministry of Education No. 81 Meishan Road Hefei Anhui 230032 China; ^4^ Reproductive Medicine Center Department of Obstetrics and Gynecology Shanghai General Hospital Shanghai Jiao Tong University School of Medicine No. 650 Xinsongjiang Road Shanghai 200080 China; ^5^ Key Laboratory for the Genetics of Developmental & Neuropsychiatric Disorders (Ministry of Education) Bio‐X Institutes School of Medicine Shanghai Jiao Tong University Shanghai 200030 China

**Keywords:** male infertility, non‐canonical splicing variant, TMF1

## Abstract

Canonical splicing variants (±2) contribute significantly to genetic disorders, yet the clinical significance of non‐canonical splicing variants (NCSVs) that occur outside of canonical splicing sites remains unknown in male infertility. A comprehensive evaluation of reported studies on hereditary male infertility revealed that the 2,404 pathogenic variants contained 120 canonical splicing variants and 32 NCSVs. Among the remaining 2,252 variants, the splicing variant analytical strategy identified 17 novel NCSVs that disrupt normal mRNA splicing from previously classified missense variants. This expands the contribution of NCSVs by 53.13% (17/32), with NCSVs accounting for 28.99% (49/169) of all the splicing variants. Moreover, thirteen positively validated NCSVs are identified in 12 of 718 idiopathic male infertility patients with negative results by conventional genetic analysis. The first pathogenic variant in the TATA element modulatory factor 1 (*TMF1*: c.2859+4A>G) results in *TMF1* exon 14 skipping and decreased progressive sperm motility and morphological abnormalities in a patient with male infertility. *Tmf1* NCSV knock‐in mice recapitulated human phenotype, showing significantly decreased sperm count, motility, ultrastructural head defects, and subfertility. This study provides the first comprehensive landscape of NCSVs in male infertility, suggesting that NCSVs may constitute a hidden etiological factor for male infertility.

## Graphical abstract

This study highlights the significance of non‐canonical splicing variants in male infertility, a factor often overlooked during the analysis of high‐throughput sequencing data. Incorporating the non‐canonical splicing variants prioritization in the genetic analysis pipeline will increase the genetic diagnosis of patients with male infertility.

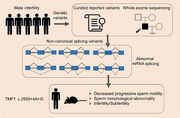

## 1 Introduction

Globally, infertility affects 8–15% couples of childbearing age and significantly affects reproductive health.^[^ ^1^, ^2^, ^3^ ^]^ Moreover, male infertility may account for 20–70% of infertility cases in couples.^[^ ^1^, ^4^ ^]^ Male infertility is characterized by a complex and multifactorial etiology, manifesting in a highly heterogeneous clinical presentation.^[^ ^5^ ^]^ Male infertility manifests as abnormalities in sperm count and/or quality. Genetic abnormalities are significant contributors to male infertility.^[^ ^3^, ^4^, ^6^ ^]^ Whole‐exome sequencing (WES) and whole‐genome sequencing (WGS), gene editing technologies, and the broad application of gene knockout or mutant mouse models have greatly facilitated the identification of pathogenic genes and variants in male infertility. Over 100 male infertility‐related genes have been identified.^[^ ^7^, ^8^ ^]^ However, these only account for a small fraction of patient phenotypes, and the genetic causes of most of the cases of male infertility remain unclear.

mRNA splicing is a key post‐transcriptional regulation in eukaryotes; over 95% genes produce mature mRNA through selective splicing.^[^ ^9^ ^]^ Moreover, 10–62% of pathogenic variants may lead to abnormal mRNA splicing.^[^ ^10^, ^11^, ^12^ ^]^ However, the guidelines established by the American College of Medical Genetics and Genomics (ACMG) primarily delineate canonical splicing variants (±2) as potential loss‐of‐function (LoF) variants.^[^ ^13^ ^]^ Emerging evidence has highlighted the significance of non‐canonical splicing variants (NCSVs) located in proximity to the donor (D) and acceptor (A) sites of the splice junction in patients with male infertility, such as potassium calcium‐activated channel subfamily U, *KCNU1*: c.1295+3A>C,^[^ ^14^ ^]^ SIX6 opposite strand transcript 1, *SIX6OS1*: c.1180‐3C>G,^[^ ^15^ ^]^ and cilia‐ and flagella‐associated protein 61, *CFAP61*: c.143+5G>A.^[^ ^16^ ^]^ Consequently, there has been a paradigm shift toward the investigation of NCSVs, leading to the discovery of numerous diagnostically relevant NCSVs.^[^ ^17^ ^]^ Moreover, the ClinGen Sequence Variant Interpretation (SVI) Working Group established an SVI Splicing Subgroup, guiding variant interpretation using splicing‐related evidence.^[^ ^18^ ^]^

Thus far, over 2000 variants have been identified in male infertility, including missense, intronic, synonymous, and frameshift variants. Some of these variants affect the mRNA splicing process that leads to male infertility, which were not detected earlier. A predicted pathogenic missense variant (cilia‐ and flagella‐associated protein 52, *CFAP52*: c.203G>T) also results in exon skipping.^[^ ^19^ ^]^ Moreover, despite the recognized potential effects of disease‐associated nucleotide variants, such as alterations in mRNA splicing, the functional impact and contribution of NCSVs to male infertility remain to be comprehensively assessed. This knowledge gap may have resulted in the inadvertent omission of clinically significant variants from consideration.

In this study, we aimed to identify the NCSVs in male infertility. Our objectives were (Figure S1, Supporting Information): 1) curation of the comprehensive sources of male infertility‐related variants from previous studies and comprehensive assessment of the impact of these variants on mRNA splicing (Figure S2, Supporting Information); 2) prioritization and validation of potential NCSVs in patients with male infertility using WES (Figure S2, Supporting Information); 3) assessment of genotype–phenotype association in patients with male infertility carrying NCSVs, including one NCSV in TATA element modulatory factor 1 (*TMF1*; the potential male infertility candidate gene), in the absence of any pathogenic variant reported in humans; 4) detailed analysis of abnormal reproductive phenotypes in humans and mouse carrying the *TMF1* NCSV.

## 2 Results

### 2.1 Prioritized Potential NCSVs from Reported Variants in Patients with Male Infertility

**Figure 1:**
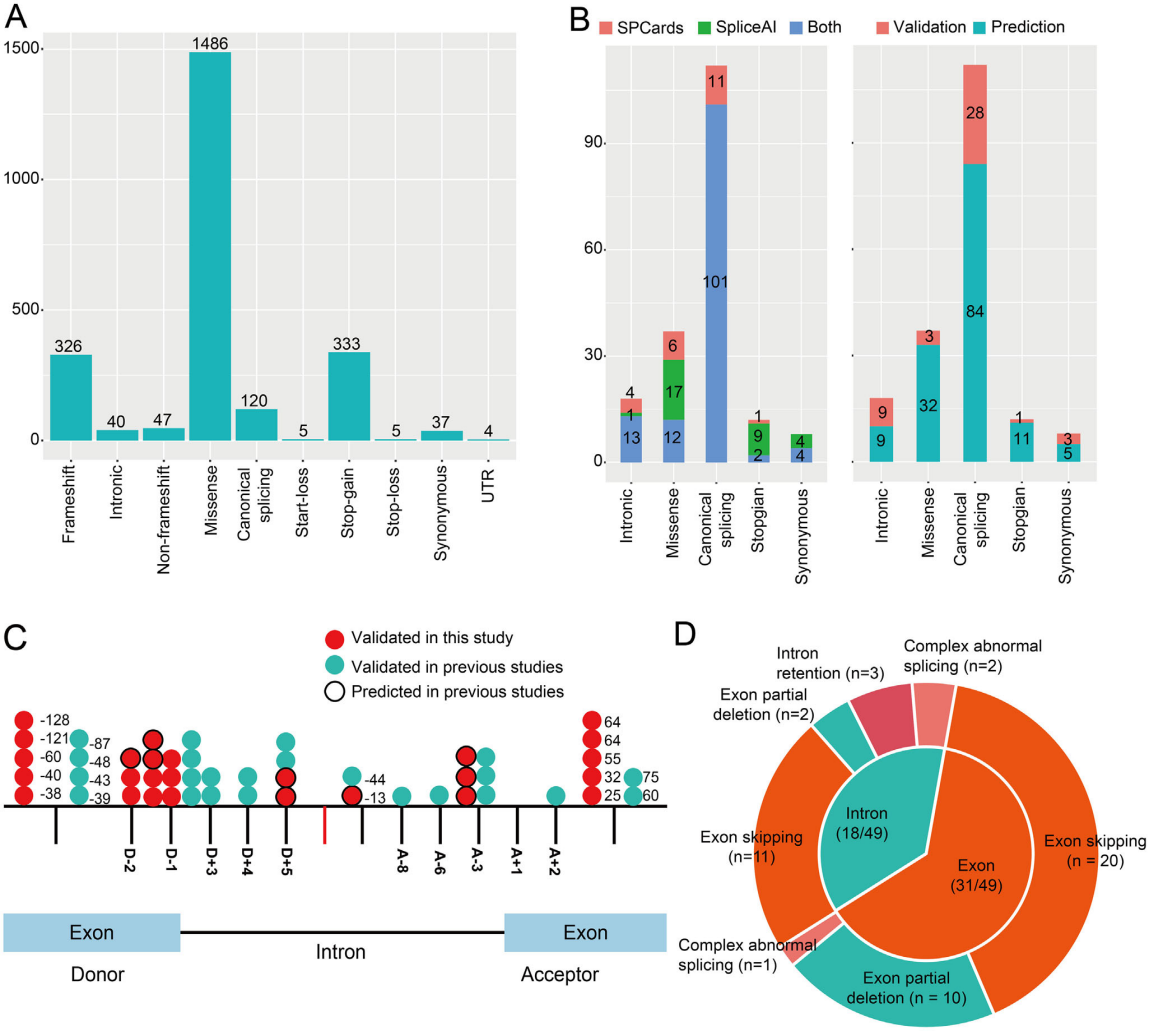
Curated variants associated with male infertility. A) Comprehensive annotation of the curated variants. These variants can be classified as frameshift, intronic, non‐frameshift, missense, canonical splicing, start‐loss, stop‐loss, stop‐gain, synonymous, and untranslated region (UTR) variants. B) SPCards (comprises many integrated methods that predict whether a specific variant is a potential splicing variant) and SpliceAI splicing variant prediction scores. C) Distribution of 49 functionally validated non‐canonical splicing variants. D) Percentage of different classifications of validation outcomes, including different abnormal splicing events in 49 functionally validated non‐canonical splicing variants.

We curated 2404 non‐redundant variants associated with male infertility (Table S1, Supporting Information). Based on comprehensive annotation, the variants could be classified into frameshift (*n* = 326), intronic (*n* = 40), non‐frameshift (*n* = 47), missense (*n* = 1486), canonical splicing (*n* = 120), start–loss (*n* = 5), stop–gain (*n* = 333), stop–loss (*n* = 5), synonymous (*n* = 37), and untranslated region (UTR) variants (*n* = 4) (**Figure** **1**A). We found 120 canonical splicing variants and 32 NCSVs (9 predicted and 23 functionally validated NCSVs in primary studies). A subset of variants may represent the pathogenic mechanisms underlying male infertility that affect mRNA splicing. We used SPCards, the splicing prediction platform that includes SpliceAI, to annotate all the curated variants. Both SPCards (which comprises many integrated methods that predict whether a specific variant is a potential splicing variant) and SpliceAI predicted high scores for canonical splicing variants (Figure S3A, Supporting Information). We then removed the canonical splicing variants, and a subset of retained variants showed high prediction scores, indicating that these variants were potential NCSVs (Figure S3A, Supporting Information). We further performed a Pearson analysis and found a significant correlation between SPCards and SpliceAI prediction scores (Pearson correlation = 0.83; P = 3.29E – 84) (Figure S3B, Supporting Information). We defined splicing variants that were predicted to be deleterious using nine or more splicing prediction methods (SPCards ≥ 9). Moreover, owing to the widespread use of SpliceAI for the prediction of splicing variants, we also designated variants with SpliceAI ≥ 0.5 as potential splicing variants (Figure S3B, Supporting Information). We prioritized 185 potential splicing variants, including intronic (*n* = 18), missense (*n* = 35), canonical (*n* = 112), stop‐gain (n = 12), and synonymous (*n* = 8) variants (Figure 1B). Most splicing variants were prioritized by both SPCards and SpliceAI (71.35%; 132/185), suggesting the reliability of prediction (Figure 1B; Table S1, Supporting Information).

### 2.2 Impact of Prioritized NCSVs on Abnormal mRNA Splicing

Previous functional analysis revealed that 23.78% (44/185) of the variants result in abnormal mRNA splicing.^[^ ^17^ ^]^ The functional effects of the remaining splicing variants, particularly the potential NCSVs, were unknown. We explored the impact of splicing variants using the minigene method. Canonical splicing and stop‐gain variants were defined as LoF variants, and the other types of potential NCSVs, including intronic, missense, and synonymous variants, were selected for functional validation (Figure 1B). We analyzed 48 potential NCSVs, including 9 predicted potential NCSVs and two functionally validated NCSVs from previous studies (Tables S1 and S2, Supporting Information). Overall, 58.33% (28/48) of the positive NCSVs, including intronic (*n* = 8), missense (*n* = 18), and synonymous (*n* = 2) variants, led to abnormal mRNA splicing (**Table** **1**). Two intronic variants (*SPINK2*: c.206‐3C>G; *MAP3K1*: c.634‐8T>A) that were validated by RT‐PCR performed using tissues of patients in primary studies.^[^ ^20^, ^21^ ^]^ The results of the validation experiments for the two intronic variants were consistent with those obtained using the minigene method. Notably, the findings of a primary study revealed that a patient with an oligozoospermia phenotype carried a compound heterozygous missense variant (centrosomal protein 192, *CEP192*: c.5750A>G; *CEP192*: c.1912C>T). One missense variant (*CEP192*: c.1912C>T) resulted in abnormal mRNA splicing (exon 14 skipping).^[^ ^22^ ^]^ Another missense variant (*CEP192*: c.5750A>G) also resulted in abnormal mRNA splicing (133 bp deletion of exon 31) (Table 1). Male infertility‐related phenotypes associated with positive NCSVs included disorders of sex development, congenital bilateral absence of the vas deferens, obstructive azoospermia, non‐obstructive azoospermia (NOA), multiple morphological abnormalities of the sperm flagella (MMAF), oligozoospermia, asthenozoospermia, and globozoospermia. In addition to the 2 verified NCSVs, we found an additional 26 functionally validated NCSVs representing a 1.13‐fold (26/23) increase over previous reports. The identified NCSVs accounted for 1.08% (26/2404) of all the previously reported variants. Based on curation and validation, we found 49 positive NCSVs (23 in previous studies and 26 in our study) in male infertility (Figure 1C). Most of NCSVs result in exon skipping or intron retention (Figure 1D). As 9 of 26 NCSVs were predicted to impact mRNA splicing, we expand the contribution of NCSVs by 53.13% (17/32), with NCSVs accounting for 28.99% (49/169) of all infertility‐associated splicing variants.

**Table 1:** Impact of reported variants on mRNA splicing in male infertility.

Classification	Position	Gene	Type	Distance	SpliceAI	SPCards	Outcome	Phenotype	Pathogenic description in the primary study	Predicted protein alteration	Fraction	PMID
Abnormal reproductive development	2:48956335:T:A	LHCGR:c.265A>T	Mis	32	0.5452	9	Exon 3 skipping	DSD	Pathogenic prediction	p.E79_I103del	25/699 (3.58%)	34338568
	5:56155534:T:A	MAP3K1: c.634‐8T>A	Intron	−8	0.8771	13	Intron 2 6bp retention	DSD	Splicing region RT‐PCR	p.A211_A212insLA	2/1512 (0.13%)	21129722
	9:99017150:C:T	HSD17B3:c.277G>A	Mis	−1	0.5969	14	Exon 3 skipping	DSD	Pathogenic prediction	p.L68Sfs*6	242/310 (78.06%)	27898418
	X:66905839:T:G	AR:c.1769‐13T>G	Intron	−13	0.9299	8	Intron 2 69bp retention	DSD	Splicing prediction	p.E589_E590insEIPEERDSGNSLSGLSTLVFVLP	23/920 (2.5%)	25248670
	X:66931411:G:A	AR:c.2053G>A	Mis	−121	0.7255	5	Exon 4 123bp deletion	DSD	Pathogenic prediction	p.A629D*	292/920 (31.74%)	37147882
Insufficient production	1:76262914:G:A	MSH4:c.244G>A	Mis	−1	0.8785	5	Exon 1 skipping	NOA	Pathogenic prediction	NA	NA	35090489
	1:109198245:C:T	HENMT1:c.226G>A	Mis	−38	0.5469	6	Exon 4 skipping	NOA	Pathogenic prediction	p.V51Gfs*36	342/393 (87.02%)	35172124
	3:52418934:G:A	DNAH1:c.8455G>A	Mis	−60	0.7429	4	Exon 53 skipping	NOA	Pathogenic prediction	p.I2775_K2838del	63/4265 (1.50%)	36017582
	3:180334459:G:A	CCDC39:c.2431C>T	Mis	25	0.6198	4	Exon 18 skipping	Oligozoospermia	Pathogenic prediction	p.C803_Q862del	60/941 (6.38%)	33005176
	4:57686748:G:C	SPINK2:c.206‐3C>G	Intron	−3	0.9482	14	Intron 2 2bp retention	NOA	Splicing region RT‐PCR	p.A69Sfs*12	65/134 (48.51%)	28554943
	7:117175465:G:C	CFTR:c.743G>C	Mis	−1	0.9534	14	Exon 6 skipping	OA (CBAVD)	Pathogenic prediction	p.G194Rfs*7	1270/1464 (86.75%)	35913788
	7:117180399:A:G	CFTR:c.1115A>G	Mis	−2	0.5325	11	Exon 8 skipping	OA (CBAVD)	Pathogenic prediction	p.T291Ifs*13	1189/1480 (80.34%)	32777524
	7:127239489:G:A	FSCN3:c.1175G>A	Mis	55	0.6137	6	Exon 5 68bp deletion	NOA	Pathogenic prediction	p.G374_R392del	18/497 (3.62%)	36572685
	9:127255309:C:T	NR5A1:c. 990G>A	Syn	−1	0.8657	15	Exon 5 skipping	NOA	Splicing prediction	p.V291_E330del	40/461 (8.68%)	35690514
	14:60923816:G:C	C14orf39:c.1180‐3C>G	Intron	−3	0.3554	14	Exon 15 skipping	NOA	Splicing region Abnormal protein localization	p.A394*	193/587 (32.88%)	33508233
	15:45258441:G:A	TERB2:c.434G>A	Mis	−1	0.9783	11	Exon 5 skipping	NOA	Pathogenic prediction	p.H117Pfs*23	103/220 (46.82%)	35690514
	16:1912054:T:A	MEIOB:c.191A>T	Mis	64	0.6563	2	Exon 4 69bp deletion	NOA	Family cosegration	p.N43_A66delinsT	24/471 (5.10%)	28206990
	18:13087149:A:G	CEP192:c.5750A>G	Mis	−128	0.9773	6	Exon 31 133bp deletion	Oligozoospermia	Pathogenic prediction	p.I1873Hfs*7	664/2537 (26.17%)	37981762
	X:69871300:C:T	TEX11:c.1483G>A	Mis	−1	0.8886	12	Exon 17 skipping	NOA	Pathogenic prediction	p.V430_K475del	46/940 (4.89%)	35413094
Insufficient release and Malfunction of male gametes	2:84932870:A:G	DNAH6:c.8726A>G	Mis	−2	0.285	12	Exon 52 skipping	MMAF	Decreased protein expression	p.K2922_R2906delinsN	85/4158 (2.04%)	37594300
	3:52428478:C:G	DNAH1:c.10627‐3C>G	Intron	−3	0.6792	17	Exon 67 skipping	MMAF	Splicing region	p.S3543Gfs*44	722/4265 (16.93%)	36510862
	10:105944769:C:T	CFAP43:c.2141+5G>A	Intron	5	0.7465	15	Intron 16 16bp retention	MMAF	Splicing prediction	p.R715Yfs*12	950/1665 (57.06%)	29449551
	10:118615261:A:G	ENO4:c.293A>G	Mis	−2	0.7707	13	Exon 2 skipping	Asthenozoospermia	Splicing prediction	p.A58_K98del	41/625 (6.4%)	37640479
	11:113230183:G:A	TTC12:c.1470G>A	Syn	−1	0.9248	15	Exon 16 skipping	MMAF	Splicing prediction	p.V491_T513del	23/680 (3.23%)	34791246
	12:63962999:C:T	DPY19L2:c.2126+5G>A	Intron	5	0.3764	15	Exon 20 skipping	Globozoospermia	Splicing prediction	p.D634Gfs*4	124/758 (16.89%)	30333325
	12:124416551:G:A	DNAH10:c.12838G>A	Mis	64	0.8015	5	Intron 74 retention and exon 75 65bp deletion	MMAF	Pathogenic prediction	p.A3744Vfs*30	212/3956 (5.36%)	34237282
	16:70884561:G:C	HYDIN:c.12441‐3C>G	Intron	−3	−	15	Exon 74 31bp deletion	Asthenozoospermia/PCD	Splicing prediction	p.R4148Sfs*4	973/5121 (19.00%)	31545650
	17:11660970:A:G	DNAH9:c.6956A>G	Mis	−40	0.7695	2	Exon 35 38bp deletion	Asthenozoospermia	Pathogenic prediction	p.G2283Afs*1	2202/4486 (49.11%)	33610189

Mis, missense; Syn, synonymous; DSD, disorders of sex development; CBAVD, congenital bilateral absence of vas deferens; OA, obstructive azoospermia; NOA, non‐obstructive azoospermia; MMAF, multiple morphological abnormalities of the sperm flagella; PCD, primary ciliary dyskinesia; Distance, distance to canonical splicing junction; SPCards, number of methods that predict the variants as potential splicing variants; Fraction, fraction of disrupted amino acid sequence of protein; NA, not available.

### 2.3 Identification of NCSVs in Patients in the Male Infertility Cohort

The general genetic analysis pipeline regards NCSVs as benign or of uncertain significance and overlooks the potential clinically relevant variants. We reanalyzed the WES data of 718 patients with male infertility without clinically relevant variants to identify potential NCSVs. We focused on the NCSVs associated with biallelic or X‐linked inheritance patterns. The biallelic inheritance pattern was associated with one homozygous or two heterozygous variants. If one heterozygous variant was a potential NCSV, we screened for other trans pathogenic variants, including canonical splicing (≤ 2 bp), stop‐gain, stop‐loss, frameshift, and deleterious missense variants, and potential NCSVs. Genes that were not related to male infertility were further removed based on the results of literature searches and functional analyses conducted previously. Sanger sequencing was performed to validate the inheritance patterns of the potential biallelic or X‐linked variants in 34 patients (Table S3, Supporting Information). The variants in 50% (17/34) of the patients had positive biallelic or X‐linked inheritance patterns, including seven homozygous variants, nine compound heterozygous variants, and one X‐linked variant (**Table** **2**; Table S3, Figure S4, Supporting Information). A patient, AY0999, carried two heterozygous NCSVs (dynein axonemal heavy chain 10, *DNAH10*: c.1436A>G; *DNAH10*: c.8113G>A). Eighteen potential NCSVs with validated inheritance patterns were identified. One potential NCSV (cytochrome P450 family 21 subfamily A member 2, *CYP21A2*: c.293‐13C>G) affects mRNA splicing, resulting in a frameshift of the amino acid sequence.^[^ ^23^ ^]^ The patient, AY2043, carrying the NCSV (*CYP21A2*: c.293‐13C>G), was diagnosed with NOA and congenital adrenal hyperplasia. Regarding the remaining 17 NCSVs, the results of the minigene experiment validated 70.59% (12/17) positive NCSVs, including six missense, two synonymous, and four intronic variants, that led to abnormal mRNA splicing (**Table** **3**; Figures S5 and S6, Supporting Information). We identified 13 NCSVs in 12 patients with oligozoospermia, NOA, MMAF, asthenospermia, or asthenozoospermia (Tables 2 and 3; Table S4, Supporting Information). These variants were present in 1.67% (12/718) of the male infertility cohort. Both heterozygous NCSVs (*DNAH10*: c.1436A>G; *DNAH10*: c.8113G>A) identified in AY0999 led to abnormal mRNA splicing (Tables 2 and 3). We rechecked the filtered variants identified using WES in the 12 patients and found no other significant clinically relevant variants (Table S5, Supporting Information). We identified compound heterozygous variants in *DNAH14* (DNAH14: c.7022G>A; DNAH14: c.6452+5G>A). *DNAH14* is associated with primary ciliary dyskinesia, an inherited disorder that might lead to structural and functional defects in motile cilia and sperm flagella.^[^ ^24^, ^25^ ^]^ An analysis performed using the Human Protein Atlas database revealed that DNAH14 exhibited testis‐specific expression, particularly in early spermatids, implying its functional involvement in spermatogenesis.

**Table 2:** Bi‐allelic or X‐linked variants of candidate genes in male infertility.

Classification	Proband ID	Phenotype	Position	Gene	Type	Genotype	1KGP	gnomAD_EAS	SIFT	PP2	MT	CADD
Insufficient production	AY0815	NOA	17:2573457:G:T	PAFAH1B1:c.400G>T	Mis	Hom	NA	NA	T	B	D	25.8
	AY1083	Oligozoospermia	18:23845279:G:A	TAF4B:c.489G>A	Syn	Hom	NA	0.0003	NA	NA	NA	23.7
	AY1291	Oligozoospermia	X:77388835:C:G	TAF9B:c.592G>C	Mis	Hemi	NA	0.000073	NA	NA	NA	24.8
	AY2043	NOA, CAH	6:32006858:C:G	CYP21A2:c.293‐13C>G	Intron	Hom	0.010	0.0021	T	NA	N	14.63
Insufficient release and malfunction of male gametes	AY1091	Asthenozoospermia	3:69075143:T:C	TMF1: c.2859+4A>G	Intron	Hom	NA	NA	NA	NA	NA	22.1
	AY0841	MMAF	1:225446838:G:A	DNAH14: c.7022G>A	Mis	Het	0.026	0.043	D	P	D	26
			1:225418858:G:A	DNAH14:c.6452+5G>A	Intron	Het	0.00060	0.0028	NA	NA	NA	10.43
	AY0796	MMAF	3:52406083:T:A	DNAH1: c.6647T>A	Mis	Het	NA	NA	D	NA	D	25.8
			3:52394439:A:G	DNAH1: c.4684A>G	Mis	Het	NA	NA	D	NA	D	28.3
	AY1274	MMAF	10:105927385:A:T	CFAP43:c. 2802T>A	Stopgain	Het	NA	NA	NA	NA	D	36
			10:105990345:T:G	CFAP43:c.319+3A>C	Intron	Het	NA	NA	NA	NA	NA	24
	AY0922	Asthenospermia	3:52420361:G:A	DNAH1: c. 8811G>A	Syn	Het	NA	NA	NA	NA	NA	22.2
			3: 52406083:T:A	DNAH1:c. 6647T>A	Mis	Het	NA	NA	D	NA	D	25.8
	AY0999	Asthenospermia	12:124272548:A:G	DNAH10:c.1436A>G	Mis	Het	NA	NA	NA	B	D	23.7
			12:124364181:G:A	DNAH10:c.8113G>A	Mis	Het	NA	NA	NA	P	D	23.7
	AY1747	Asthenozoospermia	17:7722099:G:A	DNAH2:c.10670+5G>A	Intron	Hom	NA	NA	NA	NA	NA	22.7
	AY0960	Asthenozoospermia	19:2098291:C:T	IZUMO4:c.479C>T	Mis	Het	NA	0.0013	D	P	N	23.7
			19:2097331:G:C	IZUMO4: c.298G>C	Mis	Het	NA	0.0002	D	D	D	35

Mis, missense; Syn, synonymous; Hom, homozygous; Het, heterozygous; Hemi, hemizygous; D, damaging; B, benign; P, possibly damaging. PP2, Polyphen‐2; MT, MutationTaster; NA, not available; NOA, non‐obstructive azoospermia; CAH, congenital adrenal hyperplasia; MMAF, Multiple morphological abnormalities of the sperm flagella.

**Table 3:** Functional validated non‐canonical splicing variants in the male infertility cohort.

Gene	Type	Distance	SpliceAI	SPCards	Outcome	Predicted protein alteration	Fraction
PAFAH1B1:c.400G>T	Mis	1	NA	10	Exon 6 skipping	p.V134Afs*7	276/410 (67.32%)
TAF4B:c.489G>A	Syn	−1	0.3492	12	Exon 2 skipping	p.V117Lfs*3	750/867 (86.51%)
TAF9B:c.592G>C	Mis	1	0.9007	13	Exon 6 skipping	p.A161_P197del	37/251 (14.74%)
^a)^CYP21A2:c.293‐13C>G	Intron	−13	0.7509	6	Intron 2 19bp;26bp;33bp retention	p.Y98Sfs*10; Y98Sfs*53; p.Y98F2938insPTLQPPPPPAD	387/495(78.18%);344/495(69.49%); 12/495(2.42%)
TMF1: c.2859+4A>G	Intron	4	0.3903	14	Exon 14 skipping	p.l920_G953del	33/1091(3.02%)
DNAH14:c.6452+5G>A	Intron	5	0.829	14	Exon 42 skipping	p.S2147Rfs*13	2470/4617(53.49%)
DNAH1:c.4684A>G	Mis	−2	NA	9	Exon 28 skipping	p.Y1525_R1562del	37/4265 (0.87%)
DNAH1:c.8811G>A	Syn	−1	0.4561	11	Intron 55 retention	p.E2937_V2938ins VGSRASPGIPSHMPLSSSL PSAHPLGPSSLPHPPHSSPQGPSRSMGTWVG EQKELWAEANLGGSLDMPVSCGSRDLVGRAN LSGRVPAEGCPLVLSLPHLVAGPA	105/4265(2.461%)
CFAP43:c.319+3A>C	Intron	3	0.7793	14	Exon 5 skipping	p.R195Sfs*31	1470/1665 (88.29%)
DNAH10:c. 8113G>A	Mis	1	0.1213	10	Exon 49 skipping	p.V2705_Q2760del	56/4471 (1.25%)
DNAH10:c.1436A>G	Mis	−2	0.7124	12	Intron 10 79bp retention	p.V480Ifs*17	3991/4471 (89.26%)
DNAH2:c.10670+5G>A	Intron	5	0.9686	13	Exon 69 skipping	p.G3464Pfs*14	963/4427 (21.75%)
IZUMO4:c.298G>C	Mis	−1	0.8731	14	Intron 2 retention	p.K101Yfs*74	113/214 (52.80%)

Mis, missense; Syn, synonymous; Distance, distance to canonical splicing junction site; SPCards, number of integrated methods that predict the specific variant as a potential splicing variant; Fraction, fraction of disrupted amino acid sequence of protein.

^a)^ We identified one non‐canonical splicing variant that was reported in a previous study.

### 2.4 Distribution and Classification of NCSVs

**Figure 2:**
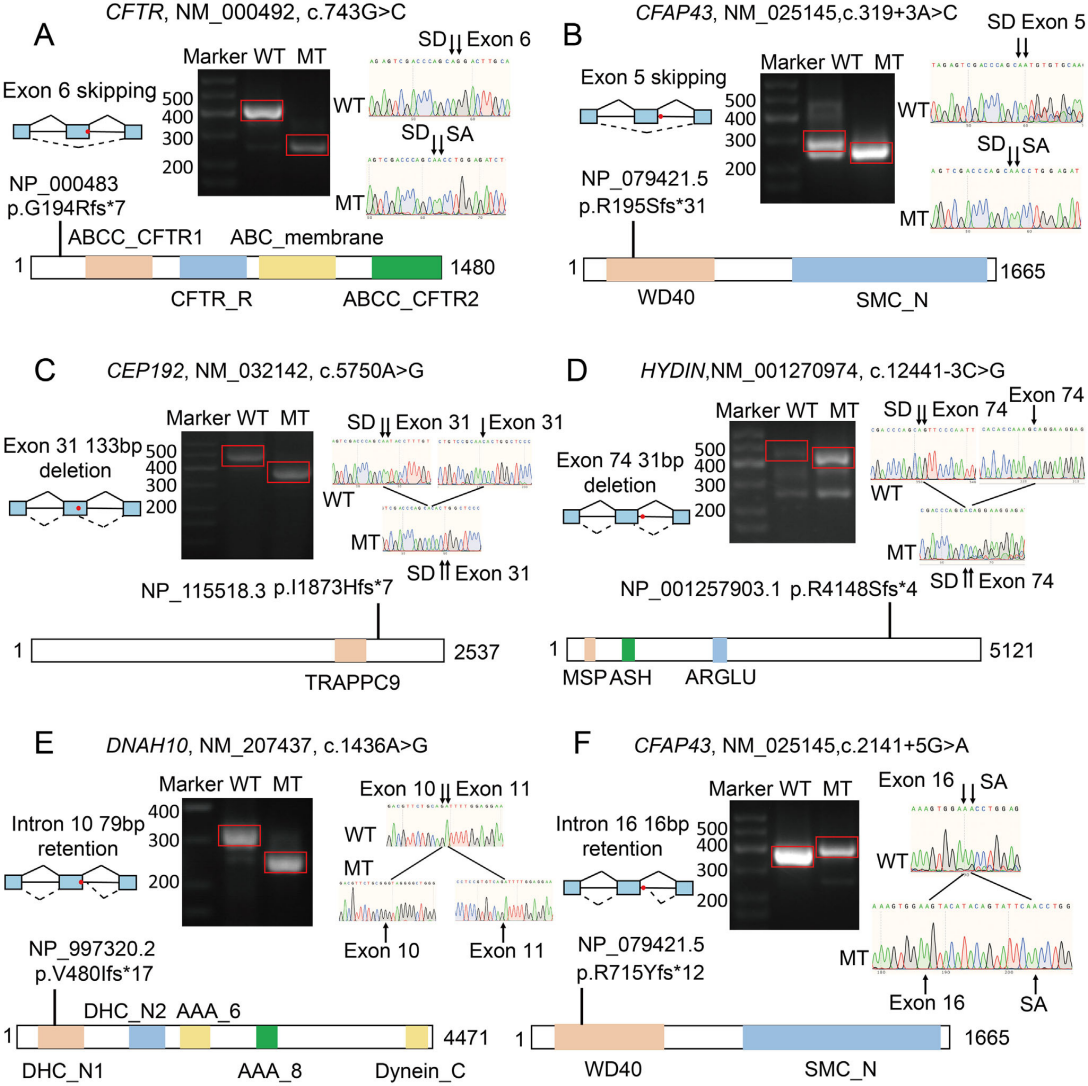
Abnormal splicing models and corresponding examples. A) Coding region variant and exon skipping; B) non‐coding region variant and exon skipping; C) coding region variant and partial exon deletion; D) non‐coding region variant and partial exon deletion; E) coding region variant and intron retention; F) non‐coding region variant and intron retention. In each section, the left side represents the abnormal splicing model, and the right side presents relevant examples.

We identified 41 positive NCSVs, including 28 previously reported variants and 13 variants identified using WES (Tables 1 and 3; Figures S6A,B, Supporting Information). The NCSVs could be categorized into six types based on whether the variant was located in the exonic or intronic region and the type of abnormal mRNA splicing (**Figure** **2**). NCSVs in both the exonic and intronic regions could lead to exon skipping (Figure 2A,B), partial exon deletion (Figure 2C,D), and intron retention (Figure 2E,F). The 27 positively validated NCSVs in the exonic region resulted in abnormal mRNA splicing, including exon skipping (*n* = 19), partial exon deletion (*n* = 5), and intron retention (*n* = 3) (Figure S6C, Supporting Information). Moreover, one exonic NCSV (*DNAH10*: c.12838G>A) led to both partial exon deletion and intron retention (Table 1; Figure S6C, Supporting Information). The 13 positive NCSVs, including one previously reported NCSV (*CYP21A2*: c.293‐13C>G) in the intronic region, led to exon skipping (*n* = 7), partial exon deletion (*n* = 1), and intron retention (*n* = 5) (Figure S6C, Supporting Information). The positive validation rates of NCSVs between the exonic and intronic regions were not significantly different (Fisher's exact test; p = 0.79; odds ratio [OR] = 1.19; 95% confidence interval [CI] = 0.36–4.24). Variants in splicing consensus regions (−3 to +8 for splicing donors; ‐12 to +2 for splicing acceptors) are likely to affect mRNA splicing. A total of 70.73% (29/41) of the identified NCSVs were in splicing consensus regions. We also identified NCSVs in the deep exonic regions. The variant (*CEP192*: c.5750A>G) was 128 bp upstream of the canonical splicing junction site, resulting in a 133‐bp deletion and frameshift in exon 31 (predicted protein alteration: p.I1873Hfs*7) (Table 1).

### 2.5 Clinical Interpretation of NCSVs

The ClinGen SVI Working Group reached a consensus that if a variant affects more than 10% of the protein product, the variant is likely to have an LoF effect and can be classified as PVS1_Strong.^[^ ^26^ ^]^ Based on the mRNA sequence changes that occurred because of abnormal splicing, we found that 58.55% (24/41) of the positive NCSVs (including one NCSV, MutS homologue 4, *MSH4*: c.244G>A, which led to exon 1 skipping) resulted in a frameshift of the amino acid sequence; 95.83% (23/24) of these NSCVs affected more than 10% of the protein (Tables 1 and 3). For example, *DNAH10*: c.1436A>G resulted in the retention of 79 bp of intron 10 (predicted protein alteration: p.V480Ifs*17), which accounted for 89.26% (3991/4471) of DNAH10 (Figure 2E). The retained NCSV, *DNAH10*: c.12838G>A, resulted in a frameshift of the amino acid sequence (predicted protein alteration: p.A3744Vfs*30) affected only 5.36% (212/3956) of the DNAH10 protein (Table 1). Overall, 41.46% (17/41) of the NCSVs did not lead to frameshift of the amino acid sequence, which accounts for 0.13–14.74% of the target protein (Tables 1 and 3). For example, the missense variant (*DNAH6*: c.8726A>G) results in exon 52 skipping, no frameshift in the amino acid sequence (p.K2922_R2906delinsN), and decreased protein expression levels.^[^ ^27^ ^]^

### 2.6 Asthenoteratozoospermia‐Related Phenotypes in a Patient Carrying Biallelic TMF1 NCSV

**Figure 3:**
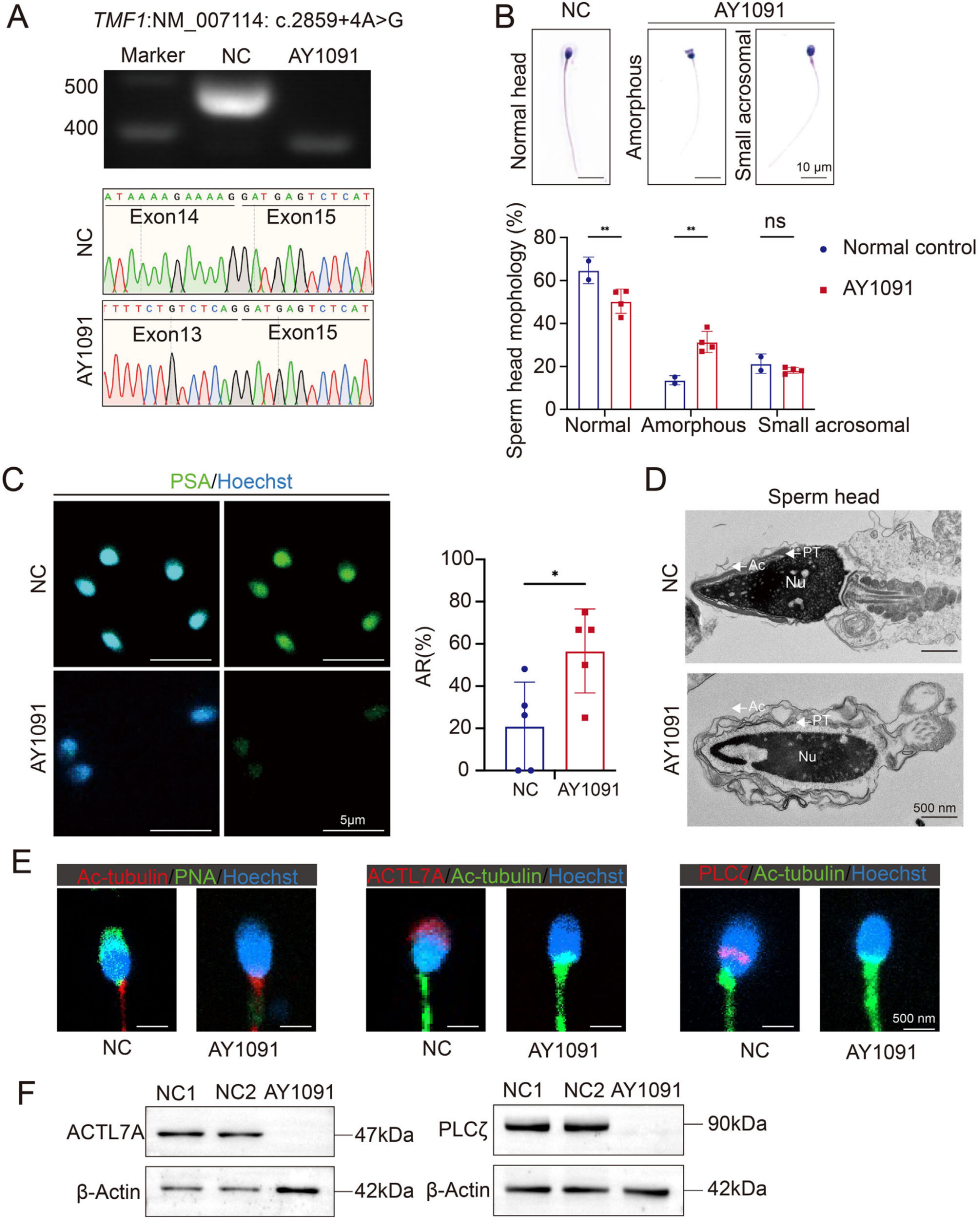
Asthenoteratozoospermia‐related phenotypes in a patient carrying a biallelic *TMF1* non‐canonical splicing variant. A) Abnormal splicing of *TMF1* was verified using Sanger sequencing in a functional experiment on in vivo splicing. NC, normal control; AY1091, patient identifier. B) Morphological analysis of haematoxylin and eosin (H&E)‐stained sperm from the *TMF1* affected patient (scale bar = 10 µm). One‐way analysis of variance (ANOVA); *p* ^**^ < 0.01. C) Spontaneous acrosome reaction test. *Pisum sativum* agglutinin‐fluorescein isothiocyanate (PSA‐FITC; green) and Hoechst (blue) were used to stain acrosomes and cell nuclei, respectively (scale bar = 5 µm). The acrosome reaction refers to the condition in which PSA‐FITC fluorescent bands are present only in the equatorial segment, or fluorescence staining is undetectable in the acrosomal area. D) Ultrastructure of the patient's sperm head. Ac, acrosome; Nu, nucleus; Pt, perinuclear theca (scale bar = 500 nm). E) Acrosomal signals were not distributed uniformly in the sperm of the patient (scale bar = 500 nm). Peanut agglutinin (PNA) labelled in the outer acrosomal membrane (green); actin‐like protein 7A (ACTL7A; red); phospholipase C zeta (PLCζ; red); acetylated microtubules (Ac‐tubulin; red/green); Hoechst (blue). F) Western blot assays of ACTL7A and PLCζ using sperm samples from two control men and the patient. β‐Actin was used as a loading control.

We identified a homozygous NCSV (*TMF1*: c.2859+4A>G) in a patient, AY1091, with asthenoteratozoospermia. RT‐PCR on the sperm of the patient found that exon 14 skipping (predicted protein alteration: p.l920_G953del) affected 3.02% (33/1091) of the amino acid sequence of the TMF1 protein (Table 2 and **Figure** **3**A). TMF1 is a Golgi‐ and microtubule‐associated protein that regulates sperm development; defects in TMF1 resulted in asthenoteratozoospermia and male infertility in a mouse model.^[^ ^28^, ^29^ ^]^ H&E staining revealed that the sperm of patient AY1091 had a significantly abnormal head morphology, as the sperm were characterized by irregularly shaped heads and small acrosomes (Figure 3B). Morphological analysis of the patient's sperm tails did not identify clinically significant abnormalities (Figure S7, Supporting Information). Sperm head abnormalities can lead to fertilization disorders.^[^ ^30^ ^]^ Therefore, we collected fresh sperm from patient AY1091 to conduct an in vitro spontaneous acrosome reaction test. The acrosome reaction rate refers to PSA‐FITC‐labelled fluorescent bands that only appear in the equatorial segment, and no fluorescence is observed in the acrosomal region. PSA‐FITC fluorescence staining of the entire acrosomal region indicated an intact acrosomal structure. The acrosome reaction rate of the sperm of patient AY1091 was higher than that of the normal control (Figure 3C). We analyzed the ultrastructure of the spermatozoa using TEM to identify abnormal sperm in patient AY1091. In contrast to the acrosomes of spermatozoa of the normal control, the sperm acrosomes of patient AY1091 were detached from the nuclear envelope and showed shedding and a dissolved form (Figure 3D). To further explore the functional abnormalities of the acrosome, we analyzed the localization of the acrosome‐related structural markers peanut agglutinin (PNA), actin‐like 7A (ACTL7A), and phospholipase C zeta (PLCζ) in the sperm of the patient by conducting immunofluorescence assays and western blot analysis. The signals of PNA, ACTL7A, and PLCζ overlapped with the outer acrosomal membranes of sperm in the normal control. However, PNA, ACTL7A, and PLCζ signals were absent in the patient's spermatozoa (Figure 3E). Western blot revealed that ACTL7A and PLCζ proteins were absent in the patient AY1091 (Figure 3F).

### 2.7 Homozygous Tmf1 NCSV Knock‐In Causes Male Infertility in Mice

**Figure 4:**
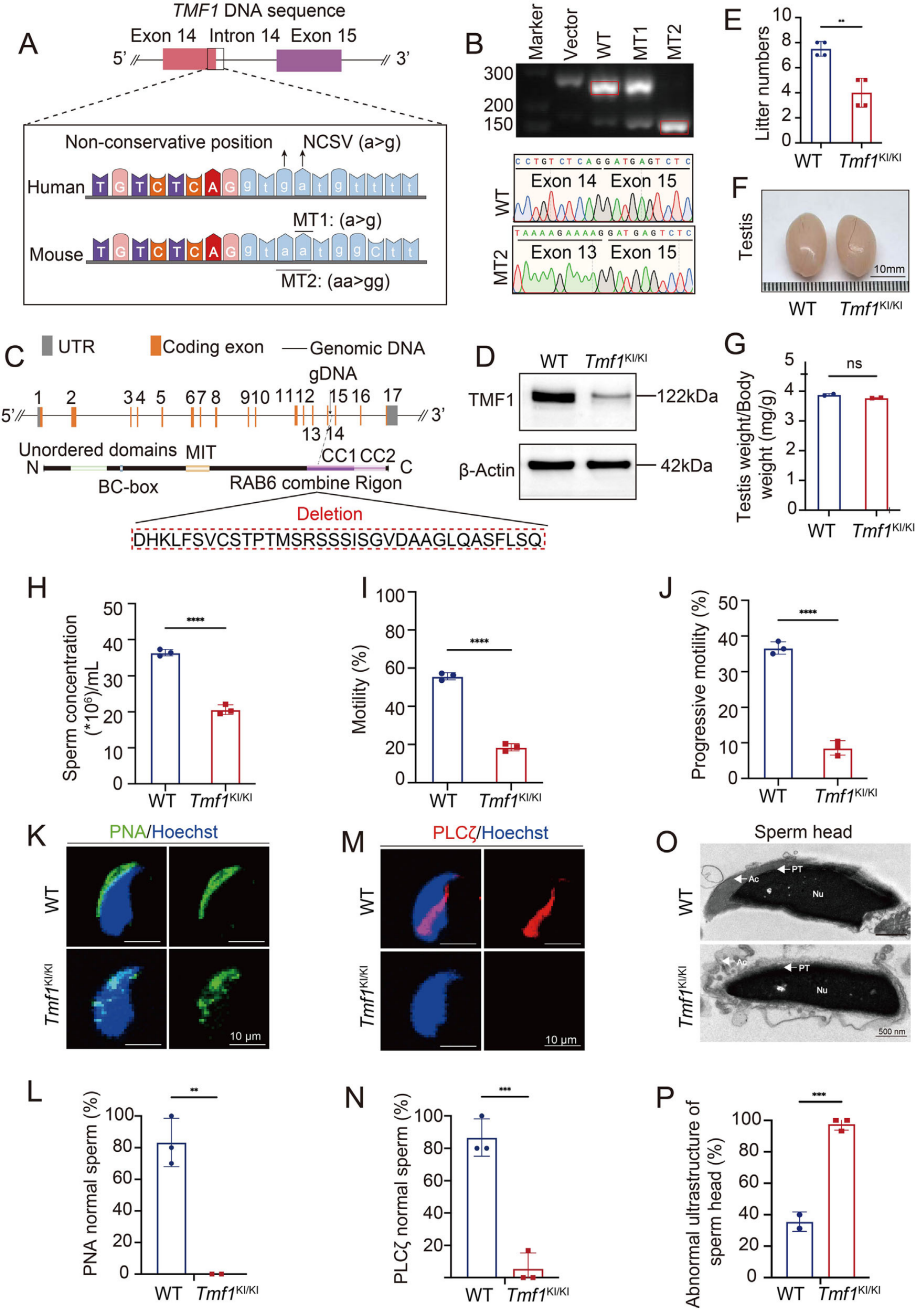
Homozygous *Tmf1* NCSV knock‐in in mice leads to acrosomal defects and male infertility. A) DNA sequences of exon 14 and intron 14 of *TMF1* in humans and mice. B) RT‐PCR analysis and Sanger sequencing revealed minigene splicing of the MT1 (*Tmf1*: c.2859+4A>G) and MT2 (*Tmf1*: c.2859+3_+4AA>GG) plasmids. C) Strategies for constructing the *Tmf1* knock‐in mouse model. D) Tmf1 protein levels were significantly decreased in the testes of *Tmf1* ^KI/KI^ mice. β‐actin was used as a loading control. E) Fertility rates of the *Tmf1* ^KI/KI^ male mice decreased. Fertility assessment experiments were performed on male wild‐type (WT; *n* = 4) mice with homozygous (*Tmf1* ^KI/KI^; *n* = 4) mutations. F) Appearance of the testes and epididymis (scale bar = 10 mm). G) Analysis of testis weight/body weight (WT, *n* = 3; *Tmf1* ^KI/KI^, *n* = 3). H–J) Epididymal sperm count and motility in *Tmf1* ^KI/KI^ mice decreased significantly (WT, *n* = 4; *Tmf1* ^KI/KI^, *n* = 4). K–N) Analysis of PNA and PLCζ signals in the acrosome, which were not distributed uniformly in sperm of the *Tmf1* ^KI/KI^ mouse (scale bar = 50 µm). PNA labelled in the outer acrosomal membrane (red); acetylated microtubules (green); Hoechst (blue). Phospholipase C zeta (PLCζ; red); acetylated microtubules (Ac‐Tubulin; green); Hoechst (blue). O, P) Ultrastructure of sperm heads from WT and *Tmf1* ^KI/KI^ mice. Ac, acrosome; Nu, nucleus; PT, perinuclear theca (scale bar = 500 nm). One‐way ANOVA; ^**^ *p* < 0.01, ^***^ *p* < 0.001, ^****^ *p* < 0.0001.

We used the CRISPR/Cas9 system to generate NCSV knock‐in mice and explored the association between *TMF1* NCSV (*TMF1*: c.2859+4A>G) and male infertility. Analysis of the *TMF1* DNA sequences of humans and mice revealed that the base sequences in humans and mice were not conserved near the NCSV, which may affect the splicing process (**Figure** **4**A). To simulate the *TMF1* splicing abnormalities observed in patient AY1091, we constructed a minigene using mouse sequences and generated two variants to perform an in vitro minigene splicing assay. For the first mutation, we generated one variant, which was the *TMF1* NCSV in patient AY1091: MT1 (*Tmf1*: c.2859+4A>G). For the second mutation, we changed the base sequence from “A” (mice) to “G” (human) at a non‐conserved position: MT2 (*Tmf1*: c.2859+3_+4AA>GG). RT‐PCR analysis and Sanger sequencing showed that the minigene splicing assay of the MT2 plasmid resulted in skipping of the entire exon 14 of *Tmf1*, similar to that observed in patient AY1091 (Figure 4B). Therefore, we constructed a *Tmf1*‐MT2 knock‐in mouse model. The knock‐in mutation was located in the CC1 domain of the *Tmf1* orthologue in mice (Figure 4C). RT‐PCR analysis revealed skipping of exon 14 of *Tmf1* in the testes of *Tmf1*‐mutated mice (*Tmf1* ^KI/KI^) (Figure S8, Supporting Information). Western blot revealed that the TMF1 protein levels in the testes of *Tmf1* ^KI/KI^ decreased significantly compared with those in the wild‐type (WT) mice (Figure 4D). To confirm the infertility of *Tmf1* ^KI/KI^ mice, four male mice with the *Tmf1* knock‐in mutation were mated with WT female mice for three months. We found that the fertility of *Tmf1* ^KI/KI^ male mice significantly decreased (Figure 4E). Testis size and weight were similar in WT and *Tmf1* ^KI/KI^ male mice (Figure 4F,G). The phenotypes of the *Tmf1* ^KI/KI^ male mouse sperm released from the cauda included a decreased sperm concentration and reduced sperm motility (Figure 4H–J).

To further examine abnormalities in the sperm of the NCSV knock‐in mice, we used PAS‐hematoxylin to stain the testes and observed normal seminiferous tubules and spermatogenesis (Figure S9A, Supporting Information). H&E‐stained epididymal sections and sperm obtained from *Tmf1* ^KI/KI^ male mice were morphologically similar to those in normal controls (Figure S9B–D, Supporting Information). We further analyzed the integrity of the sperm acrosomes by conducting immunofluorescence assays for PNA and PLCζ. In Tmf1^KI/KI^ mice, PNA labelling revealed a patchy, non‐uniform acrosomal pattern, whereas PLCζ was completely undetectable in the sperm head (Figure 4K–N). We performed TEM analysis to investigate the ultrastructure of sperm in *Tmf1* ^KI/KI^ male mice. The nuclear envelope was detached from the sperm (Figure 4O,P).

## 3 Discussion

Genetic variants significantly contribute to male infertility. Prioritized variants only account for a small fraction of patient phenotypes. Emerging evidence has highlighted the significance of NCSVs located in proximity to the donor and acceptor sites of the splice junction in patients with male infertility. However, the contribution of NCSVs to male infertility was unknown. To fill this gap in the association between mRNA splicing defects and male infertility, we used in silico prediction tools and performed experiments for functional validation to evaluate the contribution of NCSVs to male infertility.

We identified 41 positive NCSVs, comprising 28 variants previously reported in the literature and 13 variants discovered within our male infertility cohort. The pathogenicity of the 16 NCSVs was determined on the basis of missense pathogenic prediction tools in previous studies. Moreover, five of the thirteen positive NCSVs identified in our male infertility cohort can also be prioritized by missense pathogenic prediction tools. This indicates that the mechanisms for the pathogenicity prediction of variants may not be entirely accurate, particularly in the case of missense variants.^[^ ^17^ ^]^ Eight of the thirteen positive NCSVs (one missense, five intronic, and two synonymous variants) identified in our male infertility cohort were filtered out using a general genetic analysis pipeline, suggesting an essential role of additional information on variant annotation.

The splicing prediction method was effective in identifying NCSVs. We used two strategies (SPCards ≥ 9; SpliceAI ≥ 0.5) to prioritize potential NCSVs. These strategies yielded a positive validation rate of 62.12% (41 out of 66). However, 14 of 66 prioritized potential NCSVs did not impact mRNA splicing, highlighting the need for continued refinement and development of splicing prediction algorithms to improve their accuracy and reliability in identifying pathogenic variants. Splice‐altering variants might localize to deep‐intronic sequence, whereas contemporary predictors remain disproportionately focused on the splice‐junctional periphery. Consequently, genome‐wide and tissue‐resolved models of splice‐regulatory grammar are urgently required. The emergence of large‐language models such as ChatGPT has catalyzed the deployment of foundation‐scale AI in biomedicine: DeepRare,^[^ ^31^ ^]^ an agentic rare‐disease diagnostic system, furnishes fully traceable reasoning chains; EVO2^[^ ^32^ ^]^ and AlphaGenome,^[^ ^33^ ^]^ two biology‐oriented foundation models, quantitatively forecast the comprehensive molecular phenome of any variant—including aberrant splicing, transcriptional perturbation, chromatin accessibility, and transcription‐factor occupancy—thereby enabling clinically actionable interpretation at single‐nucleotide resolution.

The integrity of the sperm acrosomal structure and function is crucial for normal fertilization. Fertilization failure (FF) is one of the most common causes of male infertility. Several pathogenic genes affecting oocyte activation and sperm capacitation have been identified.^[^ ^30^, ^34^, ^35^, ^36^ ^]^ TMF1 is localized to the Golgi apparatus within spermatogenic cells. Its presence is crucial for the formation of the acrosome and the normal development of spermatids and spermatozoa in mouse models.^[^ ^29^ ^]^ However, no clinical reports have linked pathogenic variants of *TMF1* to male infertility in humans. In this study, we identified a homozygous NCSV (*TMF1*: c.2859+4A>G) in a patient AY1091 with asthenoteratozoospermia. A *TMF1* NCSV knock‐in mouse model also showed defective acrosomes, which confirms the hypothesis that NCSV (*TMF1*: c.2859+4A>G) is an FF‐related gene that plays an indispensable role in spermatogenesis. Sperm from patients with the present patient carrying the *TMF1* variant can yield good‐quality blastocysts through intracytoplasmic sperm injection (ICSI) combined with artificial oocyte activation (AOA) (Table S6, Supporting Information).

This study had several limitations. First, we predicted splicing of single‐nucleotide variants based on the results of analysis conducted using the SPCards database; we neglected insertion/deletion variants that also affect mRNA splicing. Second, we prioritized several stop‐gain variants as potential splicing variants. Stop–gain variants were defined as LoF variants in the standard pipeline and were not included in the functional validation experiment performed using minigene. For example, the variant (sodium channel alpha‐subunit type 1, *SCN1A*: c.1985C>A) resulted in both stop–gain (p.S662X) and abnormal splicing (p.R617Qfs*7).^[^ ^37^ ^]^ Third, we only included single exons or up to three exons in our minigene constructs based on the findings of a previous study,^[^ ^38^ ^]^ which led to several WT minigenes showing abnormal bands apart from the expected normal splicing patterns. Large‐scale mis‐splicing events, such as full intron retention and multi‐exon skipping, were not detected in our minigene system. We speculate that this may be due to the absence of regulatory sequences from distant regions involved in mRNA splicing from our minigene constructs.^[^ ^39^ ^]^ The SCN2A minigene requires the inclusion of five or seven exons and all the intervening introns to ensure that the WT sequence does not produce abnormal mRNA bands.^[^ ^37^ ^]^ Fourth, the minigene assay alone is insufficient to formally reclassify variants as (likely) pathogenic. Additional functional experiments are necessary to provide the evidence required for reclassification. Cas9 system‐based saturation genome editing is another strategy for accurately classifying variants.^[^ ^40^, ^41^ ^]^ Fifth, we generated knock‐in mice homozygous for NCSV (*TMF1*: c.2859+4A>G). Further analysis is required to determine the effects of other positively validated NCSVs on male infertility phenotypes.

In conclusion, variants classified as benign or of uncertain significance may contribute to aberrant splicing and the consequent gene dysfunction in patients with male infertility. Moreover, the pathogenic mechanisms of reported variants in male infertility were not fully decoded in primary studies. Some variants may be responsible for the pathogenic processes associated with male infertility because of their effects on mRNA splicing. This study highlights the significance of NCSVs in male infertility, a factor often overlooked during the analysis of high‐throughput sequencing data. Incorporating the NCSVs prioritization in the genetic analysis pipeline will increase the genetic diagnosis of patients with male infertility.

## 4 Experimental Section

### Curation of Reported Variants in Patients with Male Infertility

To identify genetic variants associated with male infertility, a comprehensive literature review on PubMed, covering the period from 1977 to 2024 was conducted. The search strategy involved combining terms related to genetic variants, such as “variant,” “mutation,” “WES,” “whole exome sequencing,” “WGS,” “whole‐genome sequencing,” and “exome,” with phenotypes pertinent to male infertility, including azoospermia, oligozoospermia, asthenozoospermia, multiple morphological abnormalities of the sperm flagella (MMAF), acephalic spermatozoa, globozoospermia, teratozoospermia, male infertility, disorders of sex development, and reproductive endocrine disorders. In the initial phase of the literature search, articles that included these search terms in their titles or abstracts were selected. The identified variants were meticulously curated by examining the abstracts, full articles, and supplementary materials. Genes that were not associated with male infertility were excluded from the analysis, based on the following criteria: 1) common variants previously used for association analysis; 2) genes only responsible for primary ciliary dyskinesia; 3) definitive benign variants that were confirmed to be “not causative” through exome sequencing in a large cohort study.^[^ ^42^ ^]^ Most studies have provided information only on changes in complementary DNA (cDNA) or amino acids in the variants. VarCards,^[^ ^43^ ^]^ the UCSC Genome Browser database,^[^ ^44^ ^]^ and TransVar^[^ ^45^ ^]^ were used to convert the cDNA or amino acid change information of variants into their corresponding genomic DNA (gDNA) positions.

### Whole‐Exome Sequencing in Male Infertility Cohort

This study was conducted at the First Affiliated Hospital of Anhui Medical University and enrolled patients diagnosed with male infertility. The subjects under investigation exhibited normal reproductive systems yet failed to attain a clinical pregnancy with their partners following 12 months of unprotected sexual intercourse. Karyotype analysis revealed normal results for all participants, and no pathogenic Y‐chromosome microdeletions were identified. Genomic DNA was isolated from the peripheral blood of these patients. Subsequently, WES and bioinformatic analyses were conducted in accordance with previously established protocols.^[^ ^46^ ^]^ In summary, the initial FASTQ data were mapped to the human reference genome (hg19 assembly) utilizing the BWA software. Following alignment, genetic variants were identified through the application of Samtools and the Genome Analysis Toolkit (GATK).

### Prioritization of Non‐Canonical Splicing Variants

Numerous studies have previously documented a significant array of pathogenic variants linked to human infertility. These variants encompass frameshift, missense, and intronic mutations, which have the potential to disrupt the alternative splicing of mRNA. ANNOVAR^[^ ^47^ ^]^ was used to conduct annotation of the reported variants and rechecked the consistency between the annotated and curated variants. Splicing prediction methods were used in SPCards to annotate reported variants and explore whether these variants affected mRNA splicing. The SPCards platform, which have been developed, incorporates 18 analytical methods that are designed to predict potential splicing variants.^[^ ^48^ ^]^ To obtain reliable results on splicing variants, variants were selected that were defined as potential splicing variants by nine or more splicing prediction methods (SPCards ≥ 9). Moreover, owing to the widespread use of SpliceAI to identify splicing variants, variants with SpliceAI scores ≥ 0.5 were also designated as potential splicing variants.^[^ ^12^ ^]^

Variants were identified using WES in the male infertility cohort. The variants were annotated using allele frequency databases, including the 1000 Genomes Project (1000G), gnomAD_exome, and gnomAD_genome, and deleterious variant prediction methods, including SIFT, PolyPhen‐2, MutationTaster, and CADD, using ANNOVAR,^[^ ^47^ ^]^ VarCards,^[^ ^43^ ^]^ and the Database for Non‐synonymous SNPs’ Functional Predictions (dbNSFP).^[^ ^49^ ^]^ Common variants, which were defined as those with an allele frequency greater than 0.01, were excluded from the analysis. Potential NCSVs were prioritized in patients with male infertility using the prediction threshold described above. If one missense or synonymous variant was prioritized by SPCards, this variant was defined as a potential NCSV. The focus was on the NCSVs associated with biallelic or X‐linked inheritance patterns. If one heterozygous variant was identified to be a potential NCSV, other trans pathogenic heterozygous variants, including canonical splicing (≤ 2 bp), stop‐gain, stop‐loss, frameshift, and deleterious missense variants, as well as potential NCSVs were screened. Missense variants predicted to be deleterious by more than three of the four prediction tools—SIFT, PolyPhen‐2, MutationTaster, and CADD (with a score > 20)—were classified as deleterious variants. dbNSFP provides prediction scores for non‐synonymous variants. If the prediction scores were not obtained for a missense variant using the four prediction tools, the variant was also retained. The GeneCards, OMIM, MGI, PubMed, and HPA expression databases were utilized to screen for variants carried by known genes and potential candidate genes associated with male infertility.

### Inheritance Pattern Validation

Sanger sequencing was conducted to confirm the inheritance patterns of biallelic or X‐linked variants identified using WES in the male infertility cohort. If the parental DNA samples were unavailable for patients carrying potential compound heterozygous variants (the distance between two variants was within 5000 bp), the homologous recombination method was used to validate the inheritance pattern; using this method, genomic DNA fragments harboring the two heterozygous variants are integrated into a vector, as described in a previous study.^[^ ^50^ ^]^

### Minigene‐Related Vector Construction

To analyze the effects of the potential NCSVs identified using WES, patients were recalled, extracted mRNA from lymphocytes in their blood or semen samples, and performed reverse transcription‐PCR (RT‐PCR; TOLOBIO, cat. no. 22107). For unavailable biological samples of patients carrying potential NCSVs identified by WES or curated in reported studies, minigenes were utilized to validate the impact of NCSVs on mRNA splicing (Figure S2, Supporting Information). The reported variants were excluded according to the subsequent criteria: 1) canonical splicing variants; 2) functionally validated NCSVs (except two that could not be validated using the RT‐PCR method, because of which the minigene method was used); 3) LoF variants, such as stop‐gain variants that were predicted to affect mRNA splicing. The minigenes contained one target exon. If the length of the flanking intron was ≈500 bp or less, the fragment was designed to encompass the alternatively spliced region containing the NCSV, as well as the adjacent introns and exons. The minigene splice assay vector was constructed based on the pcDNA3.1 vector.^[^ ^51^ ^]^ If the length of the flanking intron was greater than 500 bp, two methods were used to construct a minigene. First, coding and flanking introns (≈500 bp) regions of the target exon and flanking two exons were cloned into the pcDNA3.1 vector. Second, only the target exon and portions of the flanking introns (≈500 bp) were cloned into the exon trap splice reporter pSPL3.^[^ ^19^, ^52^ ^]^ The wild‐type and mutant minigenes were synthesized at Hefei Shicheng Biotechnology Co., Ltd. *Escherichia coli* DH5α competent cells (ToloBio cat. no. CC96102) were transformed using the vector carrying the minigene and cultured the cells in lysogeny broth. Subsequently, monoclonal colonies were isolated and confirmed the presence of the plasmid using Sanger sequencing.

### In Vitro Minigene Splicing Assay

HEK293T cells were plated into six‐well culture plates and maintained in a humidified incubator (37 °C, 5% CO_2_) until reaching 80% confluence. The cells were then transferred to serum‐free DMEM (Gibco, cat. no. 11965092) for 1–2 h. Subsequent to cell preparation, transfection was conducted using Lipofectamine 3000 reagent (Invitrogen, cat. no. l3000015), with a final plasmid DNA concentration of 0.5 µg per well. Wild‐type and mutant plasmids were transfected separately into six‐well plates. Six hours post‐transfection, the culture medium was replaced with complete DMEM medium supplemented with 10% fetal bovine serum (FBS; Gibco, cat. no. A5669801), 100 U L^−1^ penicillin, and 100 µg L^−1^ streptomycin (Biosharp, cat. no. BL505A). Transfected cells were then incubated in a 37 °C, 5% CO_2_ humidified incubator for 24–36 h to allow plasmid expression. Cells were harvested, and total RNA was extracted using TRIzol (Invitrogen, cat. no. 15596018CN) and reverse transcribed using RT‐PCR (ToloBio, cat. no. 22107). Target fragments of the wild‐type and mutant sequences were amplified by PCR; the band lengths of the mutant amplicons were compared with those of the wild‐type using 2.5% agarose gel electrophoresis (Biosharp, cat. no. BS081‐100g) and UV imaging. Gel bands of different molecular weights were excised. Sanger sequencing was performed to analyze changes in the mRNA coding region caused by abnormal splicing. A list of primers used for minigene amplification and detailed effects of positive splicing variants on mRNA splicing are provided in Table S7 (Supporting Information). Details of reagents and antibodies used in this study are provided in Table S8 (Supporting Information).

### Immunofluorescence Analysis

To assess the effects of acrosome‐related proteins on the location or expression profiles of sperm from patients and mice carrying the homozygous *TMF1* NCSV, immunofluorescence analysis was performed according to standard techniques described previously.^[^ ^53^ ^]^ In brief, freshly obtained samples of sperm were fixed with 4% paraformaldehyde (PFA) solution (Thermo Scientific Chemicals, cat. no. J19943.K2) for 4–6 h at 4 °C. PFA‐fixed sperm were adjusted to a density ensuring non‐overlapping visualization under a microscope, then evenly spotted onto slides for subsequent analysis. Subsequently, the slides were permeabilized with 0.3% Triton X‐100 (Biosharp, cat. no. BS084‐1000ml) for 30 min and blocked with 5% bovine serum albumin (BSA) in PBS at 25 °C for 1 h. Sperm samples were then incubated with primary antibodies overnight at 4 °C, washed three times with PBS (5 min per wash), and probed with Alexa Fluor 488 or 594 conjugated secondary antibodies for 1 h in the dark. Finally, labeled samples were visualized using a laser scanning confocal microscope (LSM 900; Carl Zeiss AG, Oberkochen, Germany).

### Immunoblotting

Testis tissues and sperm samples were gently rinsed twice with ice‐cold PBS to remove residual other tissue. Tissues were homogenized in cold radioimmunoprecipitation assay (RIPA) buffer (Beyotime, cat. no. P0013B) supplemented with 1× protease inhibitor cocktail (Roche, cat. no. 1836170001) and 1 mm phenylmethylsulfonyl fluoride (PMSF; Sigma–Aldrich, cat. no. 93482) using a sterile pestle or homogenizer (depending on tissue size) to ensure complete disruption. Sperm samples were lysed directly in 500 µL of the same RIPA buffer (with inhibitors) by vortexing briefly (3 × 10 s intervals) and incubating on ice for 30 min to facilitate lysis. Lysates (tissue homogenates or sperm lysates) were then centrifuged at 12000 × g for 20 min at 4 °C using a refrigerated centrifuge (Eppendorf, model 5424 R). The supernatant, containing total soluble protein, was carefully collected and transferred to a new pre‐chilled microcentrifuge tube, while the insoluble pellet was discarded. Protein extracts were denatured by heating at 100 °C for 10 min in 5× SDS sample loading buffer (Beyotime, cat. no. P0285‐15mL) to disrupt protein secondary or tertiary structures and ensure uniform migration during electrophoresis. Following denaturation, samples were centrifuged at 12000 × g for 5 min at room temperature to remove insoluble debris, and the supernatant was collected for SDS‐PAGE. SDS‐PAGE was performed using 10% polyacrylamide gels (Beyotime, cat. no. P0012A) cast with a Tris‐Glycine‐SDS running buffer (25 mm Tris, 192 mm glycine, 0.1% SDS; pH 8.3). Samples (20–30 µg total protein per lane) were loaded alongside a precision pre‐stained protein ladder (Thermo Fisher Scientific, cat. no. 26619) for molecular weight calibration. Electrophoresis was conducted at a constant voltage of 120 V for 90 min in a vertical electrophoresis system (Bio‐Rad, model PowerPac Basic) until the bromophenol blue dye front reached the bottom of the gel. For protein transfer, resolved proteins were electroblotted onto polyvinylidene difluoride (PVDF) membranes (Millipore, cat. no. IPVH85R). Post‐transfer, membranes were blocked to prevent non‐specific antibody binding using Tris‐buffered saline with Tween‐20 (TBST; 10 mm Tris‐HCl, pH 7.4, 150 mm NaCl, 0.1% Tween‐20; Biosharp, cat. nos. BL1979A/BS112–500g/BS100–1000mL) containing 5% skimmed milk powder (BD Difco, cat. no. 232100). An orbital shaker (50 rpm) was used for blocking at 25 °C for 2 h. Subsequently, membranes were probed with primary antibodies diluted in blocking buffer (1:1000 for rabbit anti‐target protein; Abcam, cat. no. ab12345) overnight at 4 °C with gentle agitation. After three 5‐min washes with TBST to remove unbound primary antibodies, membranes were incubated with secondary antibodies diluted 1:5000 in blocking buffer for 1 h at room temperature. Membranes were again washed three times with TBST (5 min per wash) to eliminate residual secondary antibodies. Finally, protein bands were visualized using enhanced chemiluminescence (ECL) detection reagent (Biosharp, cat. no. BL520A). Images were captured and analyzed using the ImageJ software (National Institutes of Health, Bethesda, MD, USA) to quantify band intensities.

### Transmission Electron Microscopy

For transmission electron microscopy (TEM) analysis, human and mouse sperm samples were processed as follows: small tissue fragments (1–2 mm^3^) were trimmed from collected sperm samples using a Leica EMUC7 ultramicrotome to facilitate fixative penetration and resin embedding. Trimmed samples were immediately fixed in 0.1 m sodium cacodylate buffer (pH 7.4; Electron Microscopy Sciences, cat. no. 11652) containing 2.5% glutaraldehyde (Electron Microscopy Sciences, cat. no. 16220) and 3% paraformaldehyde (Electron Microscopy Sciences, cat. no. 15710) at 4 °C for 12–16 h to preserve ultrastructural integrity. Post‐fixation, samples were rinsed three times with 0.1 m cacodylate buffer (10 min per wash) to remove excess fixative, then dehydrated through a graded ethanol series (30%, 50%, 70%, 90%, 100%; 15 min per concentration at room temperature) and infiltrated with a 1:1 mixture of 100% ethanol and propylene oxide (Electron Microscopy Sciences, cat. no. 20410) for 1 h, followed by three changes of pure propylene oxide over 2 h. Infiltrated samples were embedded in Epon 812 resin (Electron Microscopy Sciences, cat. no. 14120) Ultrathin sections (50–70 nm thick) were cut using a diamond knife (Diatome, cat. no. 33510) on a Leica Ultracut E microtome (cat. no. 14000), collected onto 200‐mesh copper grids (Electron Microscopy Sciences, cat. no. 12100), and post‐stained: 2% uranyl acetate (Ted Pella, cat. no. 19481) for 15 min to enhance contrast, followed by Reynolds’ lead citrate (Ted Pella, cat. no. 19468) for 5 min to visualize intracellular structures. Stained grids were rinsed with deionized water, air‐dried, and imaged using a Tecnai G^2^ Spirit TEM (Thermo Fisher Scientific, cat. no. 067005).

### Spontaneous Acrosome Reaction Test

For sperm sample processing, 0.2 mL of raw semen was transferred to a conical tube and mixed with 5 mL of physiological saline. The resulting suspension was centrifuged at 1000 × g for 10 min to pellet sperm cells; the supernatant was carefully aspirated, leaving 0.2–0.4 mL of concentrated sperm pellet. To enhance sperm dispersion, the pellet was gently resuspended in 1.2 mL of BWW culture medium, and the tube was tilted to a 45° angle for incubation at 37 °C for 1 h. Following incubation, the tube was returned to an upright position, and 1 mL of the uppermost solution was carefully pipetted into a new conical tube. This aliquot was then mixed with 5 mL of physiological saline, subjected to a second centrifugation (1000 × g for 10 min), and the supernatant was discarded to retain a 0.2–0.4 mL sperm pellet. For smear preparation, 5 µL of the resuspended sperm suspension was spread evenly onto a clean glass slide, air‐dried, and fixed in 95% ethanol for 30 min. The fixed smear was then incubated with 2 µL of Pisum sativum agglutinin‐fluorescein isothiocyanate (PSA‐FITC) at 2–8 °C for 1–18 h to label sperm acrosomes. After three washes with phosphate‐buffered saline (PBS) to remove unbound fluorophore, the slide was mounted with a glass coverslip. Acrosome morphology was visualized using a Zeiss LSM 900 confocal microscope (Carl Zeiss AG, Oberkochen, Germany) with excitation at 450–490 nm and emission detected through a FITC‐specific filter set.

### Generation of *Tmf1* Non‐Canonical Splicing Variant in Mice

The CRISPR/Cas9 gene‐editing system was used to construct a mouse model harboring an NCSV of *Tmf1*. The sequence at the +3 position of this gene was not conserved between humans (G) and mice (A). To mimic the effect of this NCSV on the mRNA, a base change from A to G was introduced at the +3 position in mice. CRISPR/Cas9‐mediated gene editing was performed to introduce the *Tmf1* NCSV: *Tmf1*: c.2859+3_+4AA>GG. A single guide RNA (sgRNA) with the sequence 5′‐ GTCCTTCCTGTCTCAGGTAATGG‐3′ targeting the *Tmf1*: c.2859+3_+4AA>GG locus was cloned into a pUC57 expression vector, transcribed in vitro, and purified. The synthesized single‐stranded oligodeoxyribonucleotides contained the *TMF1*: c.2859+3_+4AA>GG: CACGTTCCAGCTCTATAAGTGGAGTCGATGCTGCAGGGCTGCAAGCGTCCTTCCTGTCTCAGGT(AA>GG)TGGCTTACGTGTGGTTCTTACATGCGTTTAGTTCTTACCTTGAGTAATGCTAAAGGAAACATGC. The sgRNA, single‐stranded oligodeoxyribonucleotides, and Cas9 mRNA were mixed and subsequently microinjected into the fertilized oocytes of C57BL/6 mice. The resulting embryos were transferred, and then mice were born. Sanger sequencing was performed to confirm the presence of variants in the offspring.

### Mating Test

Four 8–10‐week‐old homozygous male mice and eight wild‐type female mice were selected. Males and females were co‐housed in cages at a ratio of one homozygous male to two wild‐type females. Vaginal plugs were checked every morning. Females with vaginal plugs were separated and monitored for gestational duration and the number of pups they were birthing. The fertility assessment was conducted over a minimum duration of three months. Calculate the average number of newborn mice per cage (number of newborn mice in the cage/number of female mice in the cage).

### Sperm Concentration and Motility Analysis

After 3–4 days of abstinence, mature sperm were obtained by masturbation for human sperm release. For mouse sperm release, both sides of the cauda epididymis were dissected from 8‐week‐old mice (*n* = 4). To facilitate sperm release, three longitudinal incisions (1–2 mm in length) were made in the surface of each cauda epididymis using Ophthalmic scissors. The incised epididymides were then transferred to a 1.5 mL microcentrifuge tube containing 500 µL of pre‐warmed (37 °C) sterile phosphate‐buffered saline (PBS; Gibco, cat. no. 10010023). The tube was incubated at 37 °C with gentle agitation for 10–20 min to allow sperm to swim out of the epididymal tubules into the surrounding medium. A 10‐µL aliquot of the sperm release fluid was collected, and sperm concentration and the percentage of motility‐impaired sperm were determined using a computer‐assisted sperm analysis (CASA) system (MICROPTIC, SCA‐H‐01).

### Morphological Analyses of Testes and Sperm

Freshly isolated spermatozoa were fixed in 4% paraformaldehyde (PFA; Thermo Scientific Chemicals, cat. no. J19943.K2) for 30 min at 37 °C to preserve cellular structure. For morphological analysis, fixed sperm were stained with hematoxylin and eosin (HE; Biosharp, cat. no. BL700A) using a standard protocol optimized for spermatozoa. The percentage of sperm with head or tail malformations was quantified by examining a minimum of 200 spermatozoa per mouse under a brightfield microscope (Zeiss, Axio Imager M2) at 400× magnification, with abnormality criteria defined as: head defects (e.g., misshapen acrosome, double heads) or tail defects (e.g., bent, coiled, or absent flagella). For histological analysis of testes and epididymides, tissues were dissected immediately after sperm collection, fixed in Bouin's solution (Sigma‐Aldrich, cat. no. HT10132) at 4 °C overnight, and stored in 70% ethanol until processing. Tissues were dehydrated through a graded ethanol series (70%, 80%, 90%, 95%, 100%; 1 h per concentration), cleared in xylene (2 changes, 1 h each), and embedded in paraffin wax (Leica, cat. no. 14020) using an automated tissue processor (Leica, ASP300S). Serial 5‐µm‐thick sections were cut using a Leica RM2235 microtome and collected onto slides. Deparaffinization was performed in xylene (3 changes, 5 min each), followed by rehydration through descending ethanol concentrations (100%, 95%, 90%, 80%, 70%; 5 min per concentration). HE staining was executed using a standard protocol (hematoxylin immersion for 5 min, differentiation with 1% hydrochloric acid‐alcohol, counterstaining with eosin for 3 min). For periodic acid‐Schiff (PAS) staining, treated with Schiff's reagent (Solarbio, cat. no. G1008‐20ML) for 15 min, and counterstained with hematoxylin. Stained sections were dehydrated, cleared, and mounted with neutral balsam. All sections were visualized using a Zeiss LSM 900 confocal laser scanning microscope (equipped with a Plan‐Apochromat 20×/0.8 M27 objective) and imaged with the Zen Black software (Zeiss). Digital images were captured at 200×magnification for morphological analysis, with representative fields selected for publication.

### Data Analysis and Statistics

Data are presented as mean ± standard deviation (SD)/standard error of the mean (SEM). Statistical significance of differences between group results was determined using a two‐sided Student's t‐test and a two‐tailed Mann‐Whitney U‐test, performed with GraphPad Prism version 8.0. A p‐value threshold of *p* < 0.05, *p* < 0.01, *p* < 0.001, and *p* < 0.0001 was considered statistically significant.

### Ethics Approval Statement

This study was approved by the Clinical Ethics Committee of the First Affiliated Hospital of Anhui Medical University (No. 2024458). Experiments with mice were conducted according to institutional guidelines for animal ethics and approved by the Anhui Medical University Experimental Animal Welfare and Ethics Committee (No. LLSC20241311).

## Conflict of Interest

The authors declare no conflict of interest.

## Author Contributions

K.L., Y.Ch., D.T., Y.S., X.Ha., H.G., and N.Z. contributed equally to this work. X.He. did conceptualization and design of the study. K.L., Y.Ch., Y.S., and X.Ha. did literature search. K.L., Y.Ch., and Y.S. did bioinformatic analysis. Y.Ch., Y.S., and X.Ha. did minigene assay. D.T., H.G., Z.D., G.W., Y.G., C.X., Q.S., B.S., Y.X., H.W., J. W., Y.Ca., and X.He. collected clinical information. Y.Ch., Y.S., X.Ha., N.Z., R.G., R.H., Z.D., Z.Y., and M.L. did human and mouse related experiments. K.L., Y.Ch. and X.He. wrote the manuscript. All the authors were involved in data interpretation and manuscript drafting.

## Supporting information



Supporting Information

Supplemental Table 1

## Data Availability

ANNOVAR (https://annovar.openbioinformatics.org/en/latest/), GeneCards (https://www.genecards.org/), Human Protein Atlas (https://www.proteinatlas.org/), OMIM (https://www.omim.org/), PubMed (https://pubmed.ncbi.nlm.nih.gov/), SPCards (http://www.genemed.tech/spcards/), SpliceAI (https://github.com/Illumina/SpliceAI), UCSC (https://genome.ucsc.edu/)
