# Supplementary material for: Mapping the Non‐Canonical Splicing Variants: Decrypting the Hidden Genetic Architecture of Idiopathic Male Infertility
Source: Adv Sci (Weinh). 2025 Oct 30;13(9):e15512. doi: 10.1002/advs.202515512 (PMC12904016; doi:10.1002/advs.202515512)
Supplement: Supplementary file 1 — Supporting Information [file ADVS-13-e15512-s002.pdf]

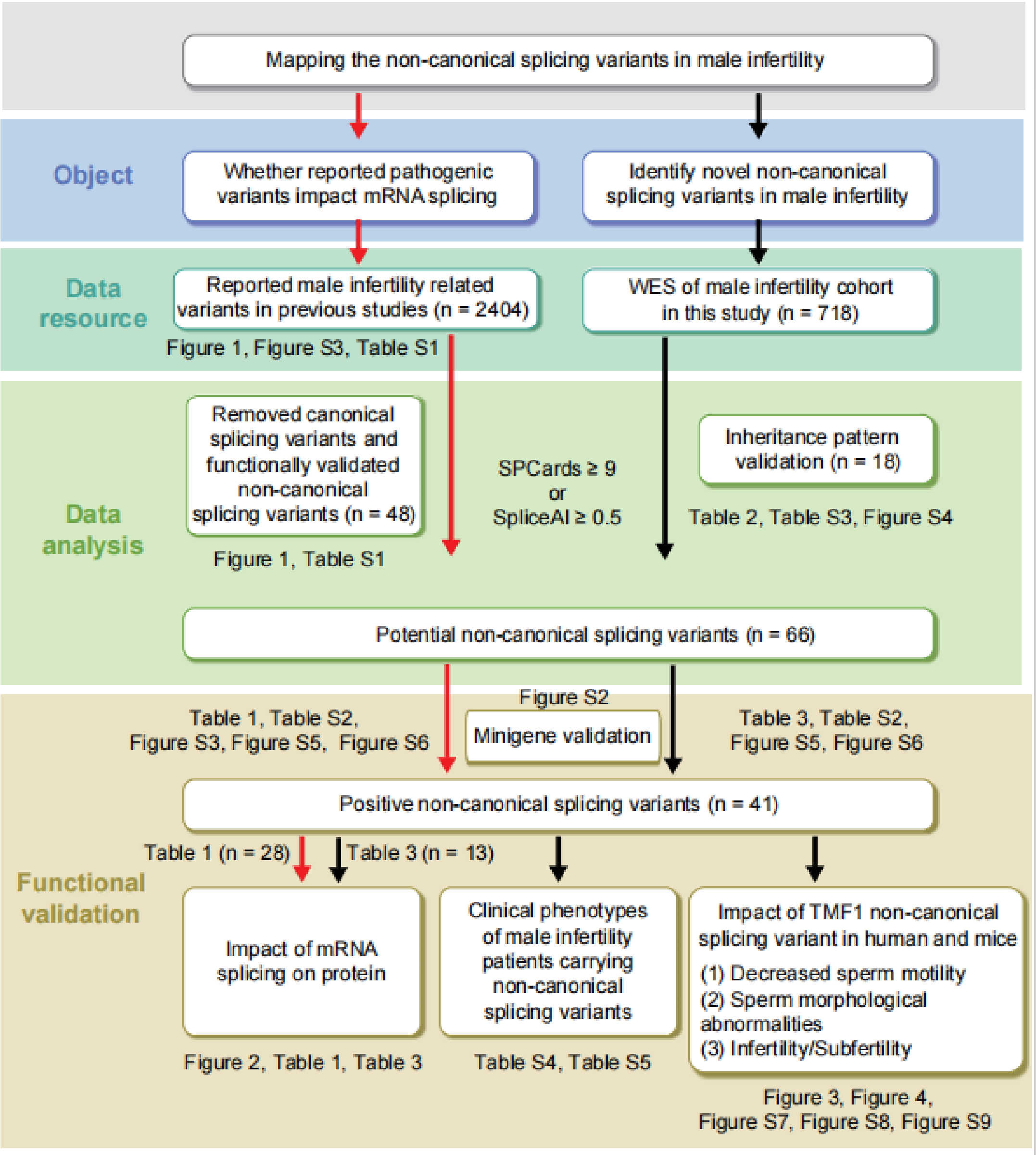

Figure S1. Study workflow

Our study objectives were (1) curation of the comprehensive sources of male infertility-related variants from previous studies and comprehensive assessment of the impact of these variants on mRNA splicing; (2) prioritization and validation of potential non-canonical splicing variants in patients with male infertility using WES; (3) assessment of genotype–phenotype association in patients with male infertility carrying non-canonical splicing variants; (4) detailed analysis of abnormal reproductive phenotypes in humans and mouse carrying the TMF1 non-canonical splicing variants. Red arrows indicate reanalyzed from literature, and black arrows indicate new experiments.

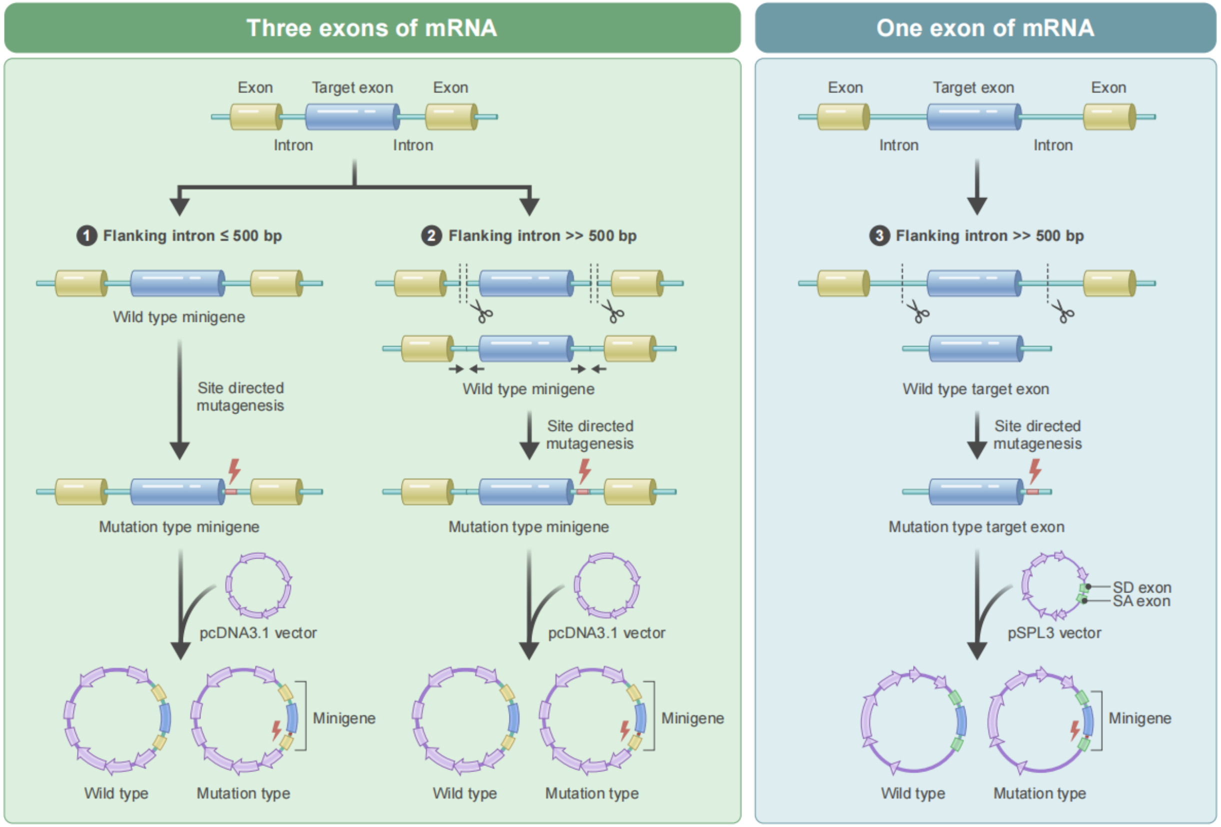

Figure S2. Minigene construction

Minigenes contained one target exon. 1) If the length of the flanking intron was approximately 500 bp or less, the fragment was designed to encompass the alternatively spliced region containing the NCSV, as well as the adjacent introns and exons. The minigene splice assay vector was constructed based on the pcDNA3.1 vector. If the length of the flanking intron was greater than 500 bp. 2) We cloned coding and flanking introns (approximately 500 bp) regions of the target exon and flanking two exons into pcDNA3.1 vector. 3) We only cloned the target exon and portions of the flanking introns (~500 bp) into the exon trap splice reporter pSPL3.

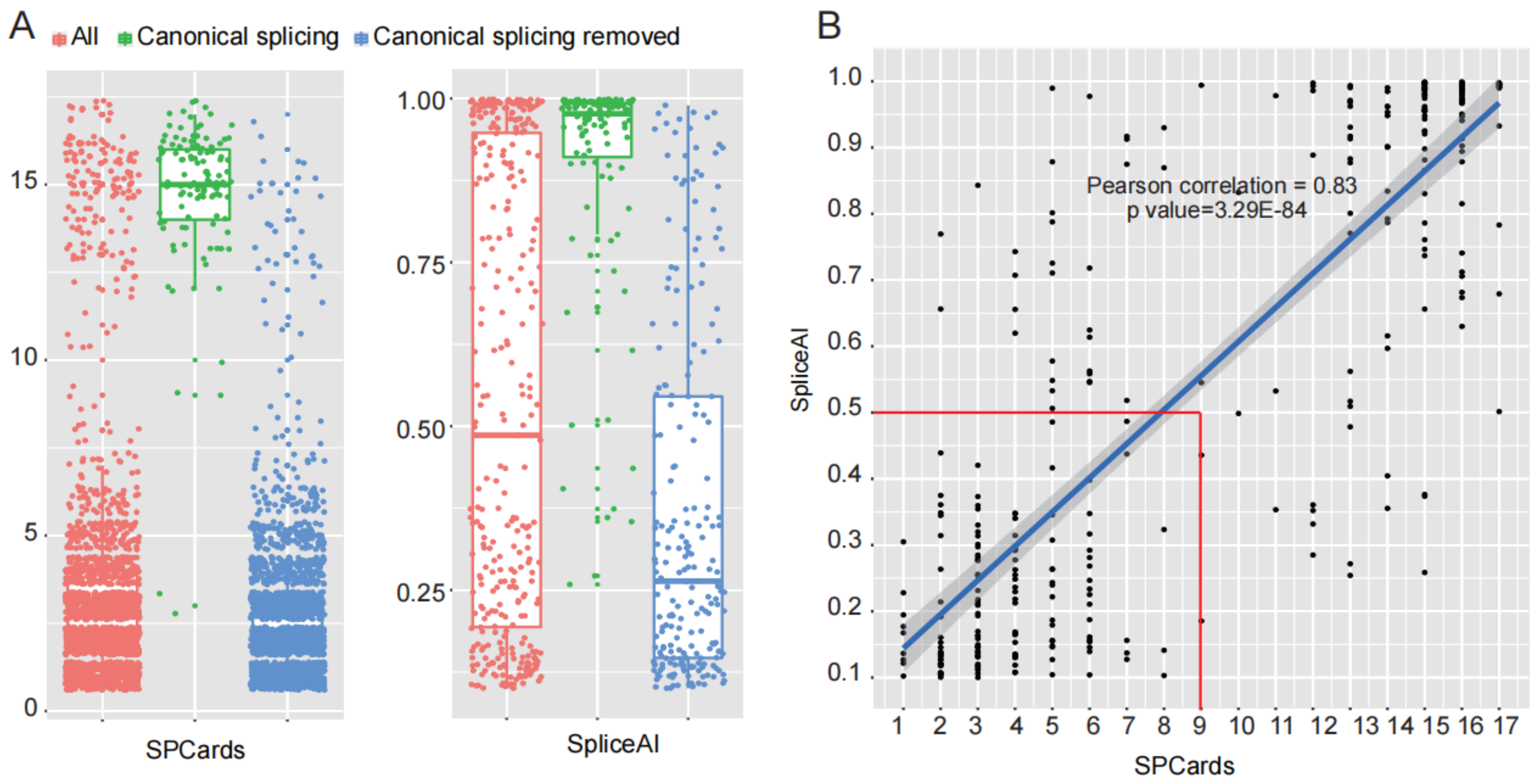

Figure S3. Splicing variant prediction

A, SPCards (comprises many integrated methods that predict whether a specific variant is a potential splicing variant) and SpliceAI splicing variant prediction scores. B, Analysis of Pearson correlation between SPCards and SpliceAI prediction scores. We defined splicing variants that were predicted to be deleterious using nine or more splicing prediction methods (SPCards $\geq$ 9) and SpliceAI $\geq$ 0.5.

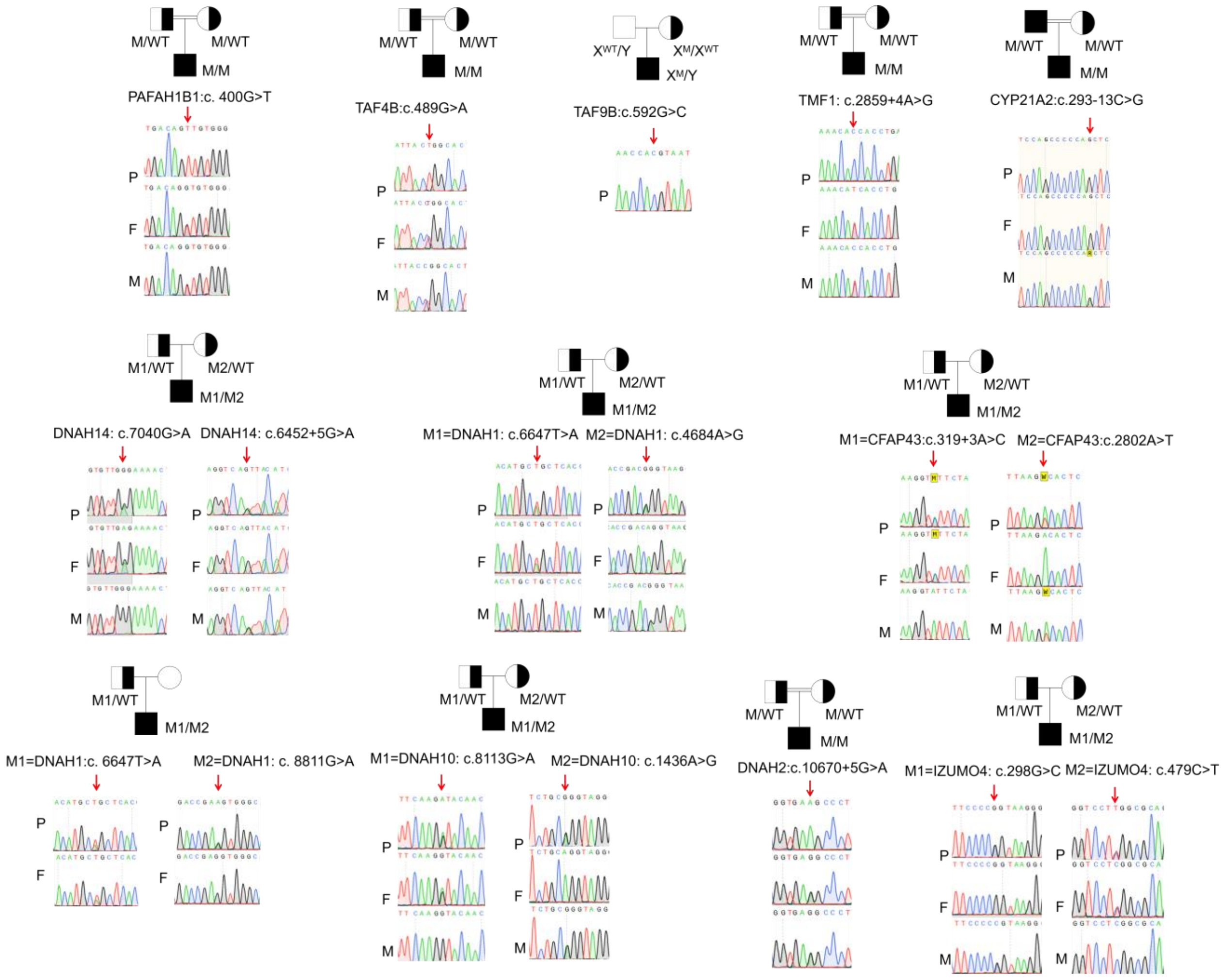

Figure S4. Pedigrees of the families with genes involved in male infertility

WT, wild type; M, mutation; P, patient; F, father; M, mother

**Abnormal splicing**		14	NR5A1,NM_004959,exon5,c. 990G>A,p.E330E	28	DNAH9,NM_001372,exon35,c.6956A>G, p.D2319G
1	LHCGR,NM_000233,exon3,c.265A>T,p. I89L	15	C14orf39, NM_174978, intron 14, c.1180-3C>G	29	PAFAH1B1,NM_000430,exon6,c.400G>T,p.V134L
2	MAP3K1, NM_005921, intron2, c.634-8T>A	16	TERB2, NM_152448, exon5, c.434G>A, p.S145N	30	TMF1,NM_007114.2, intron14, c.2859+4A>G
3	HSD17B3, NM_000197, exon3, c.277G>A, p.E93K	17	MEIOB,NM_001163560,exon4,c.191A>T ,p.N64I	31	TAF4B,NM_001293725,exon2,c.489G>A ,p.P163P
4	AR, NM_001348064, intron2, c.1769-13T>G	18	CEP192,NM_032142,exon31,c. 5750A>G,p.N1917S	32	TAF9B,NM_015975,exon6,c.592G>C,p. V198L
5	AR,NM_000044,c. 2053G>A,p.V685I	19	TEX11,NM_031276,exon17,c.1483G>A, p.A495T	33	DNAH14,NM_001373, intron42,c.6452+5G>A
6	MSH4,NM_002440,exon1,c.244G>A,p.G 82S	20	DNAH6,NM_001370,exon52,c.8726A>G, p.Q2909R	34	DNAH1,NM_015512,exon28,c.4684A>G, p.R1562G
7	HENMT1,NM_001102592,exon4,c.226G >A,p.G76R	21	DNAH1,NM_015512, intron66,c.10627-3C>G	35	CFAP43,NM_025145, intron5,c.319+3A>C
8	DNAH1,NM_015512,exon53,c.8455G>A ,p.G2819R	22	CFAP43,NM_025145, intron16,c.2141+5G>A	36	DNAH1, NM_015512, exon55, c. 8811G>A,p.E2937E
9	CCDC39,NM_181426,exon18,c. 2431C>T,p.R811C	23	ENO4,NM_001242699,exon2,c.293A>G, p.K98R	37	DNAH10,NM_207437,exon49,c.8113G>A,p.V2705I
10	SPINK2, NM_001271718, intron2, c.206-3C>G	24	TTC12,NM_001352037,exon16,c.1470G >A,p.E490E	38	DNAH10,NM_207437,exon10,c.1436A>G,p.Q479R
11	CFTR, NM_000492, exon6, c. 743G>C, p.R248T	25	DPY19L2,NM_173812,intron20,c.2126+5 G>A	39	DNAH2,NM_020877,intron69,c.10670+5 G>A
12	CFTR,NM_000492,exon8,c.1115A>G,p. Q372R	26	DNAH10,NM_207437,exon75,c.12838G >A,p.G4280R	40	IZUMO4,NM_001031735,exon2,c. 298G>C,p.G100R
13	FSCN3, NM_020369, exon5, c.1175G>A, p.R392Q	27	HYDIN, NM_001270974, intron 73,c.12441-3C>G		

Figure S5. Minigene validation outcomes for predicted splicing variants
A summary of validated non-canonical splicing variants including the results of agarose gel electrophoresis and Sanger sequencing.

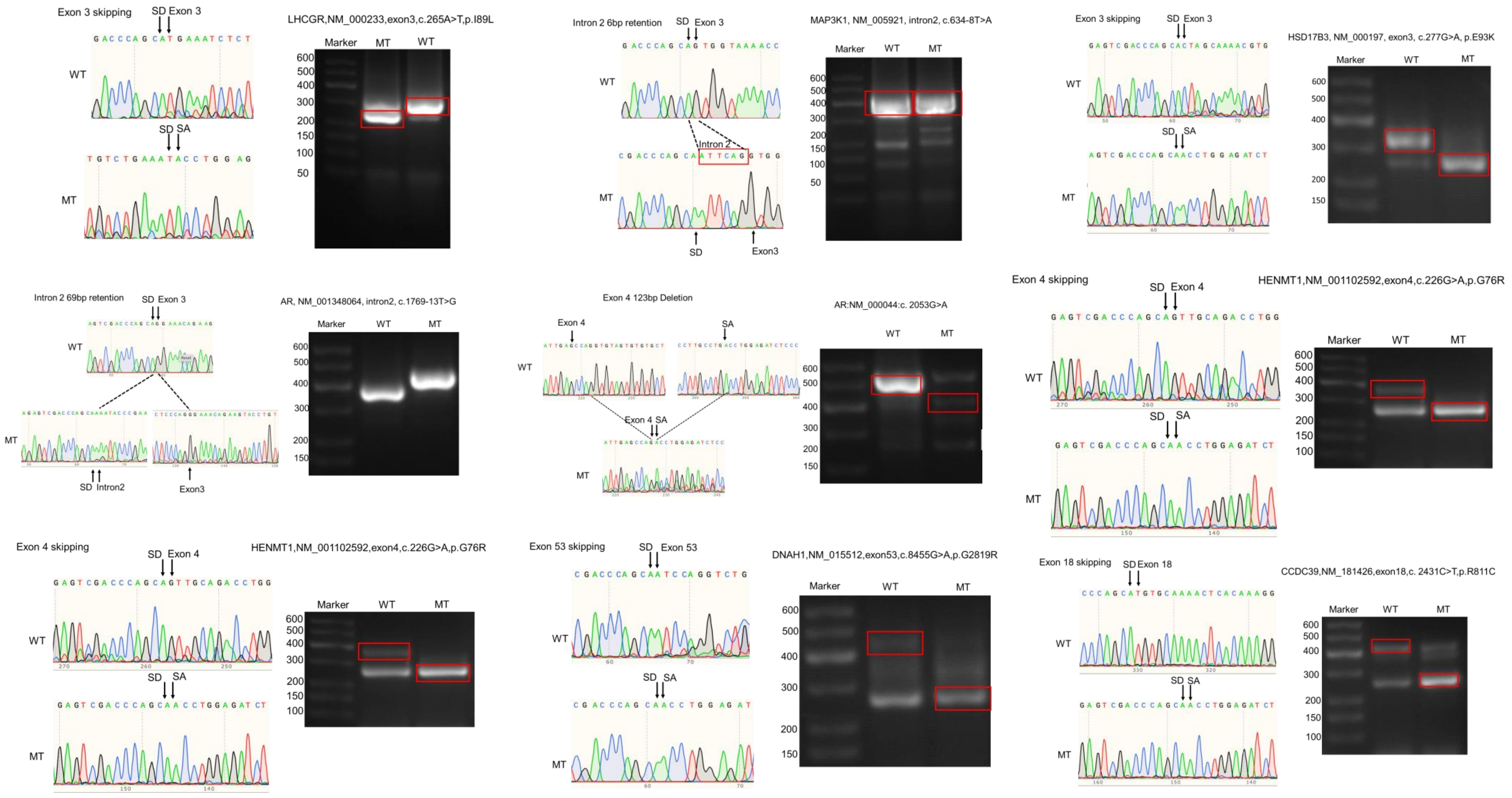

Figure S5. Minigene validation outcomes for predicted splicing variants

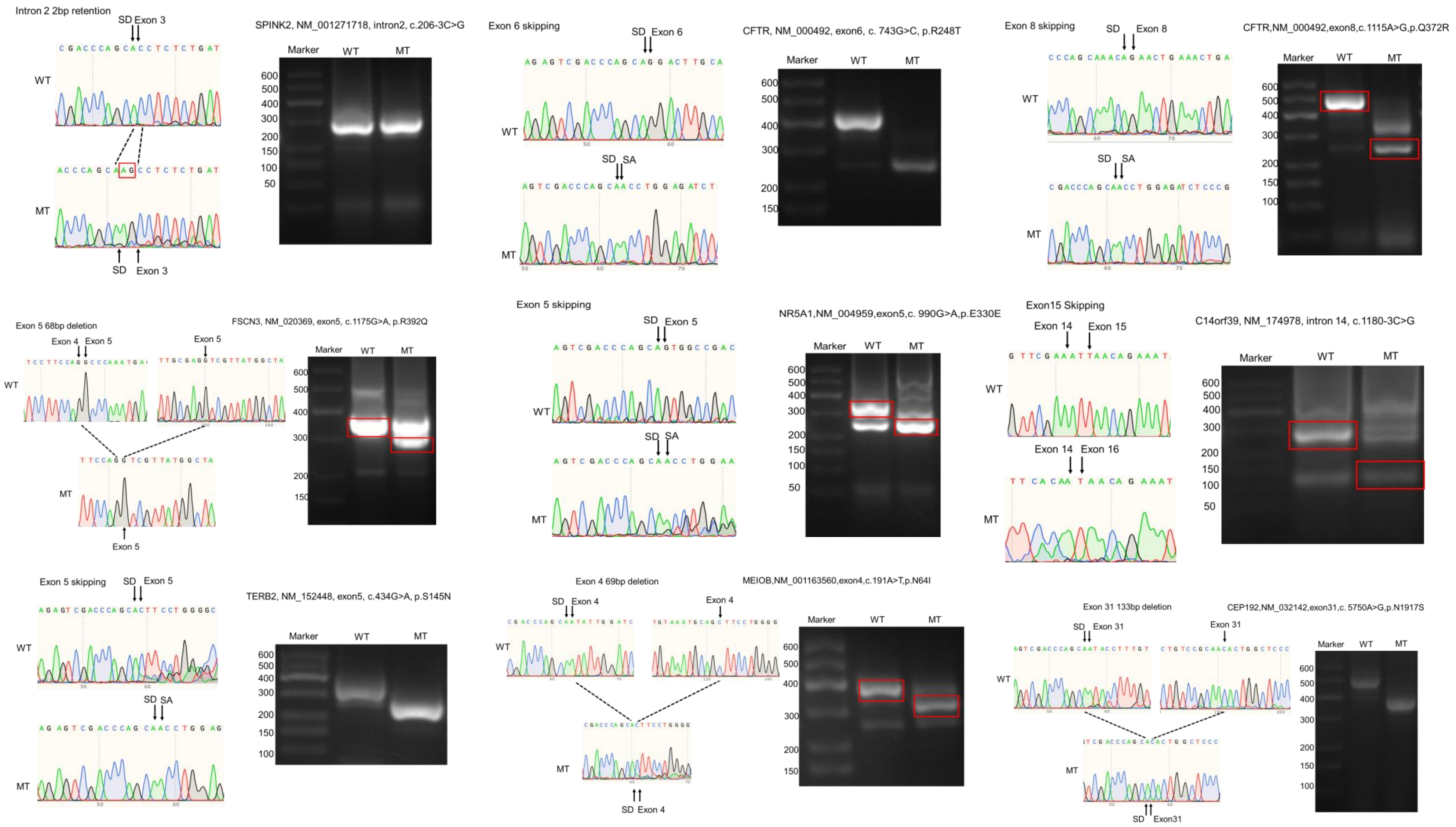

Figure S5. Minigene validation outcomes for predicted splicing variants

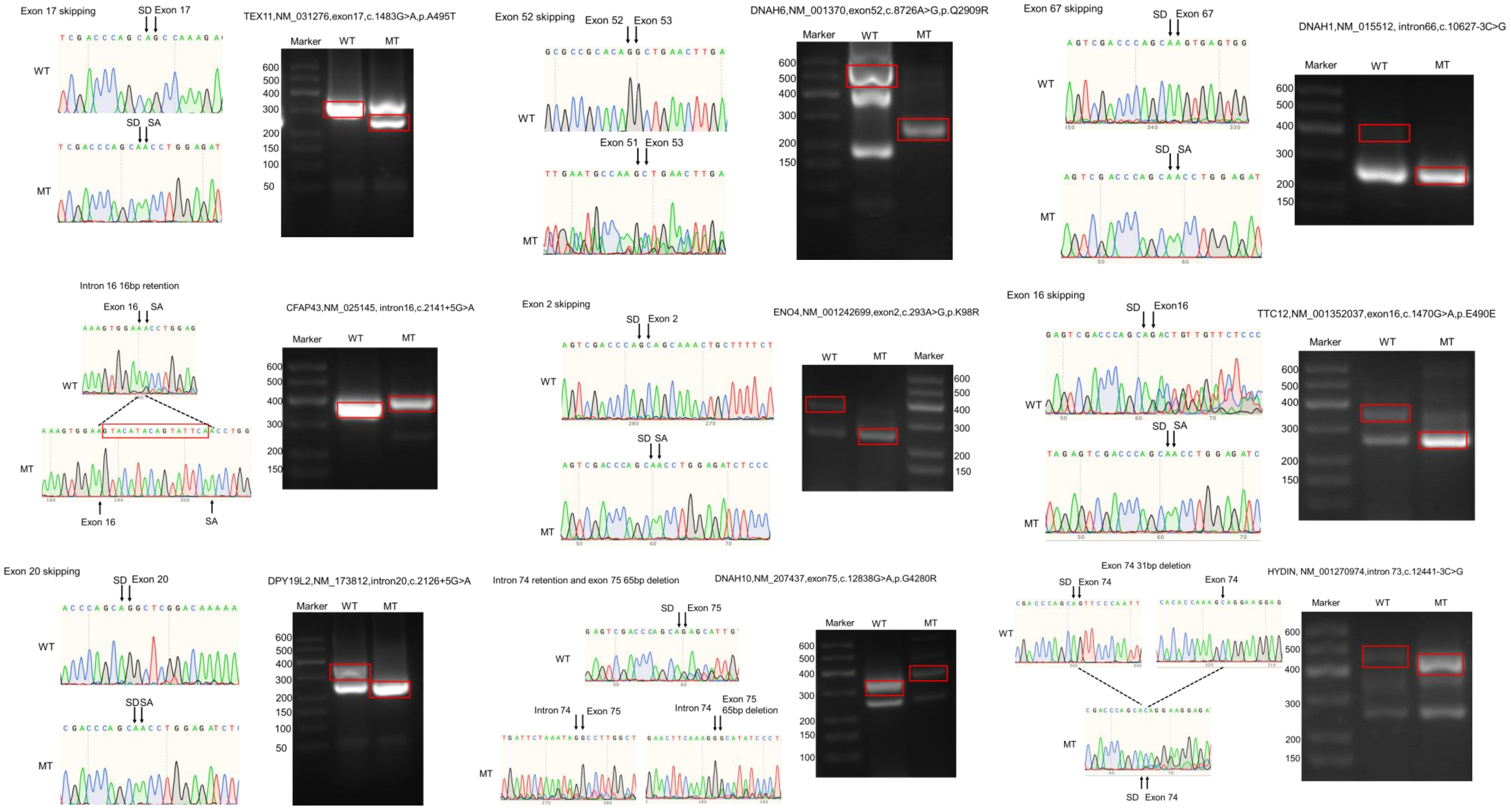

Figure S5. Minigene validation outcomes for predicted splicing variants

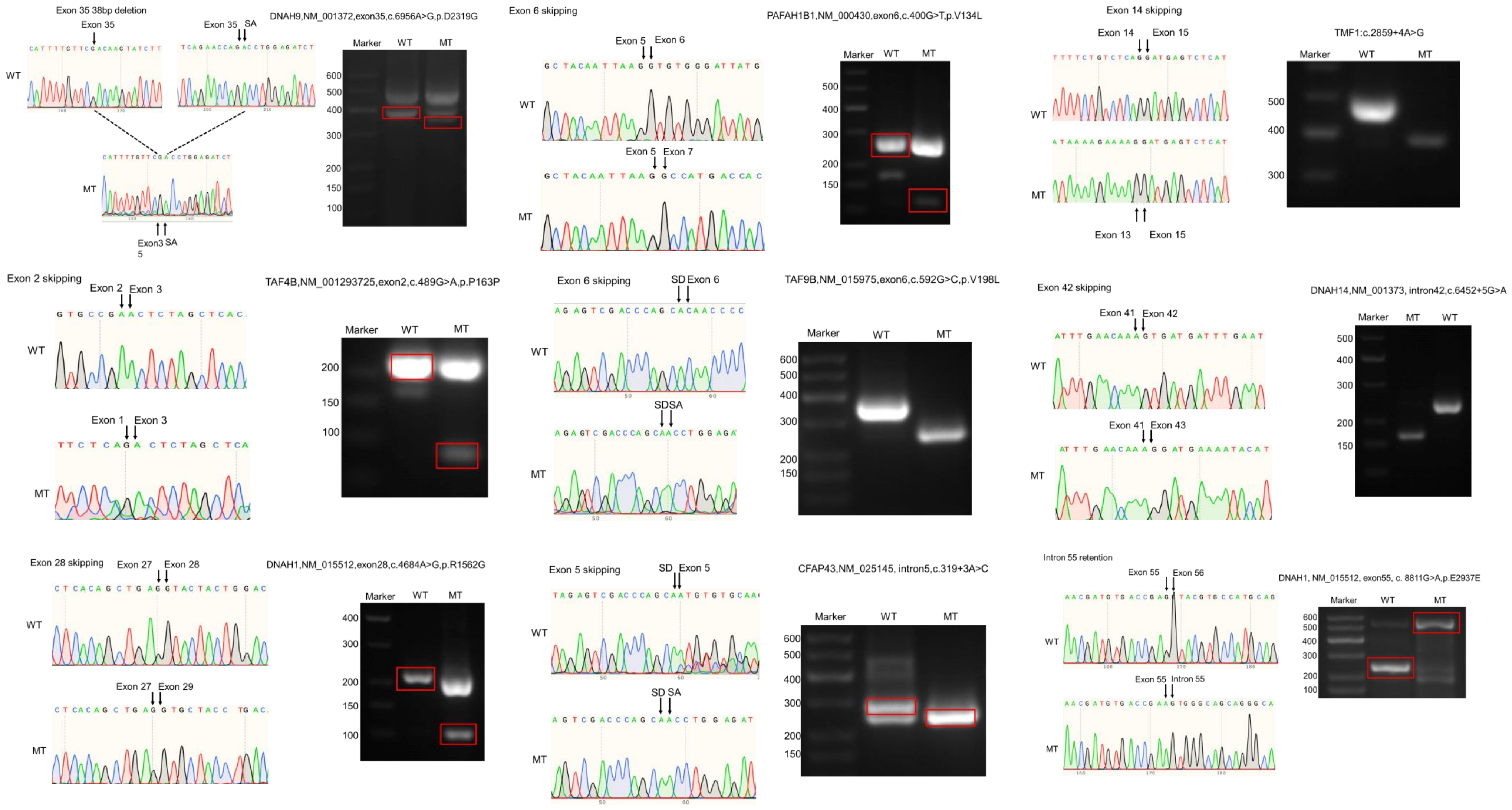

Figure S5. Minigene validation outcomes for predicted splicing variants

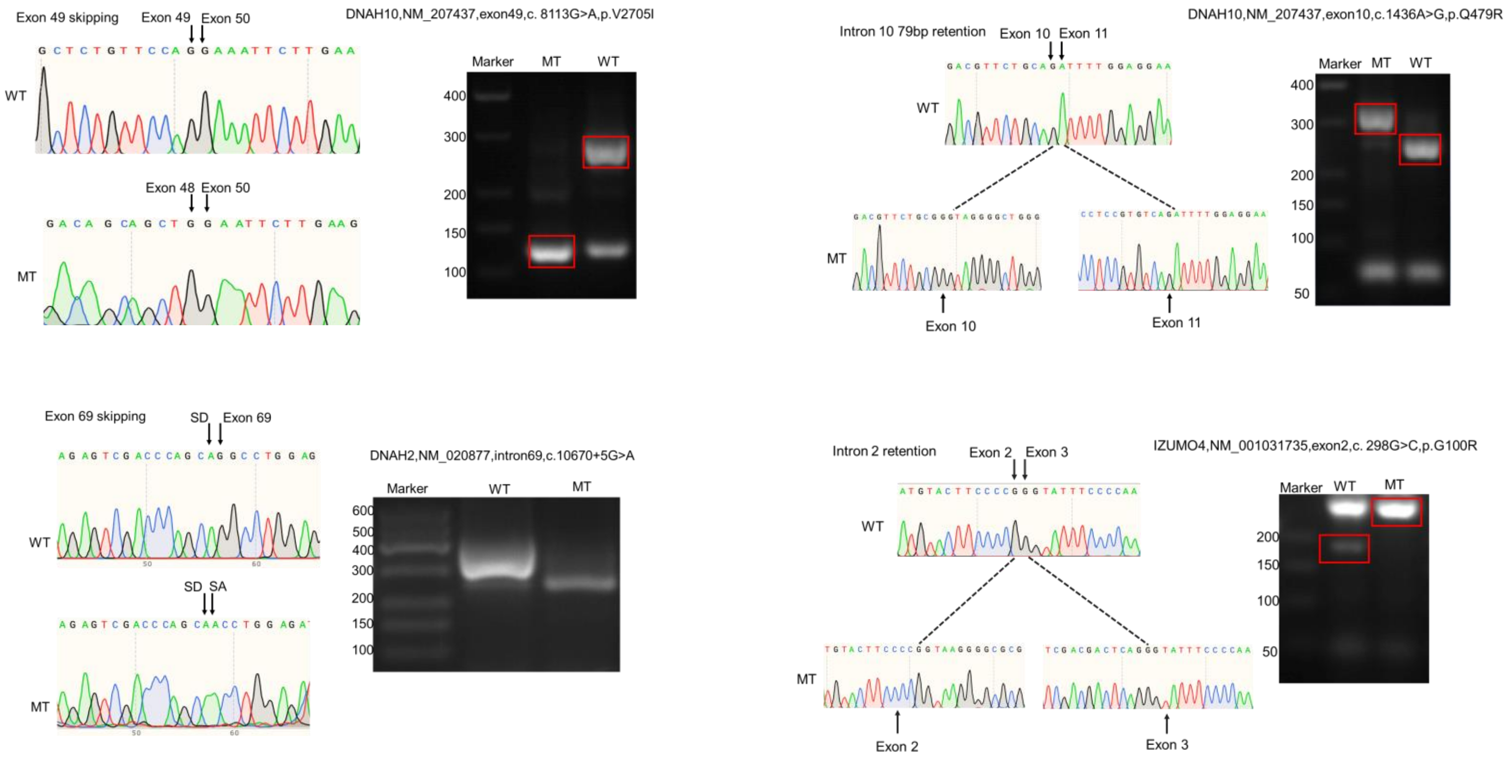

Figure S5. Minigene validation outcomes for predicted splicing variants

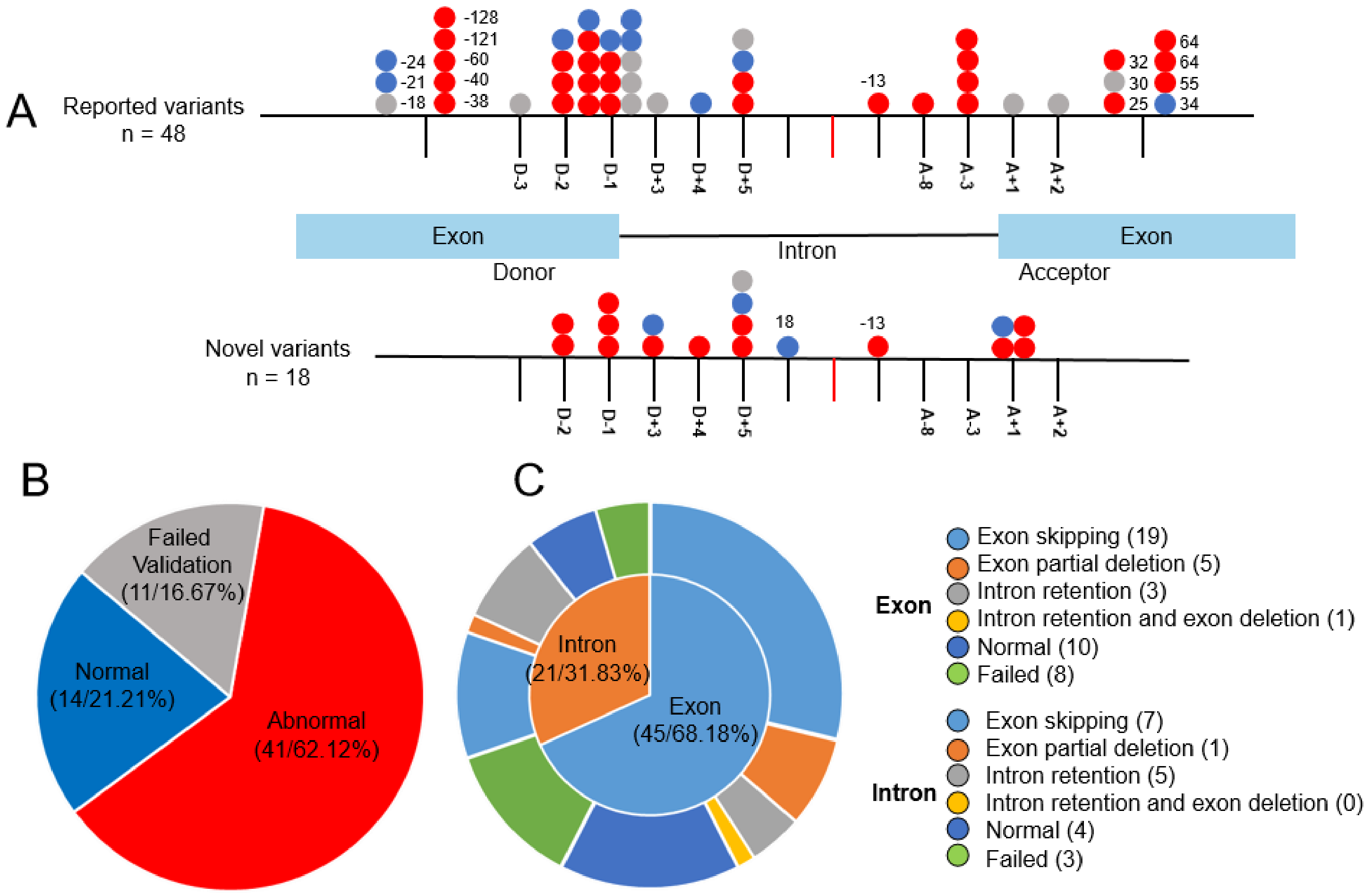

Figure S6. Effects of minigene-validated variants for splicing

A, Distribution and outcome of validated putative splicing variants. “A” and “D” indicate splicing acceptor and donor, respectively. Red, blue, and grey indicate abnormal, normal, and failed variants, respectively. D-3, D-2, D-1, D+3, D+4, D+5, A-8, A-3, A+1 and A+2 indicate variants within the splicing consensus regions. B,Validation rate of splicing variants. C, Percentage of different classifications of validation outcomes including different abnormal splicing events.

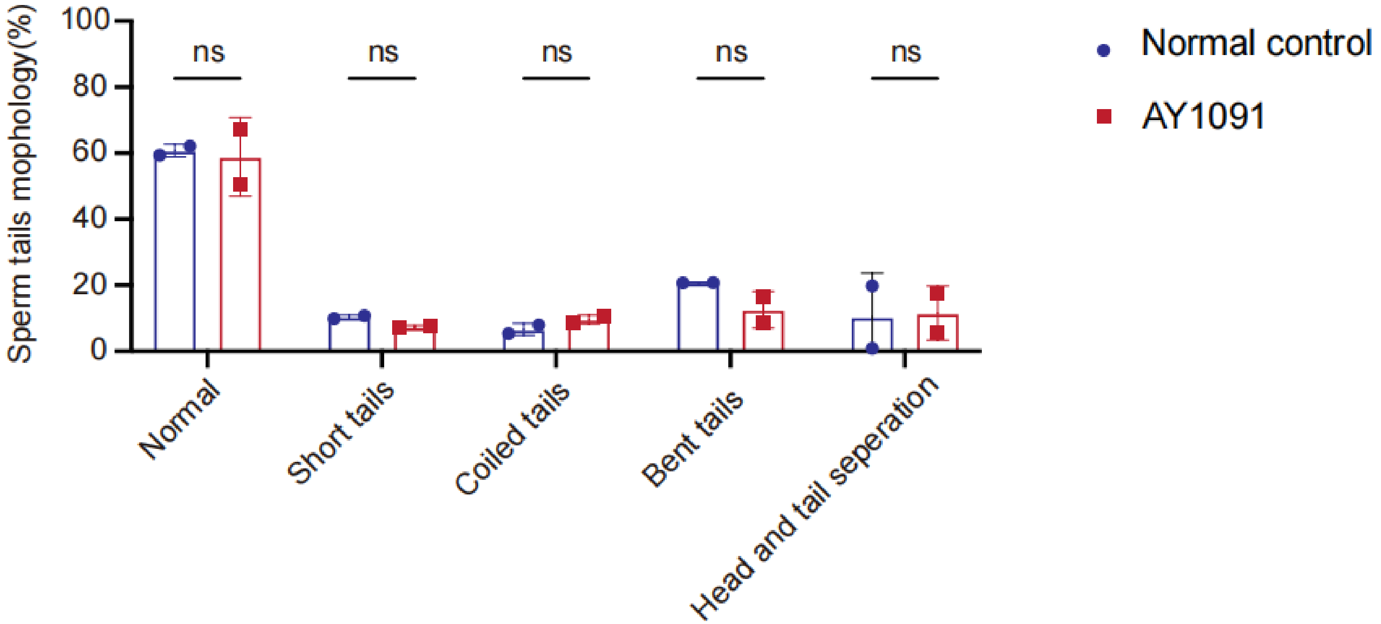

Figure S7. Sperm tail morphological analysis of an infertile patient with asthenoteratozoospermia carrying homozygous NCSV in *TMF1*.

Sperm tail morphological analysis in the TMF1 affected patient under H&E staining. One-way ANOVA, ns, not significance.

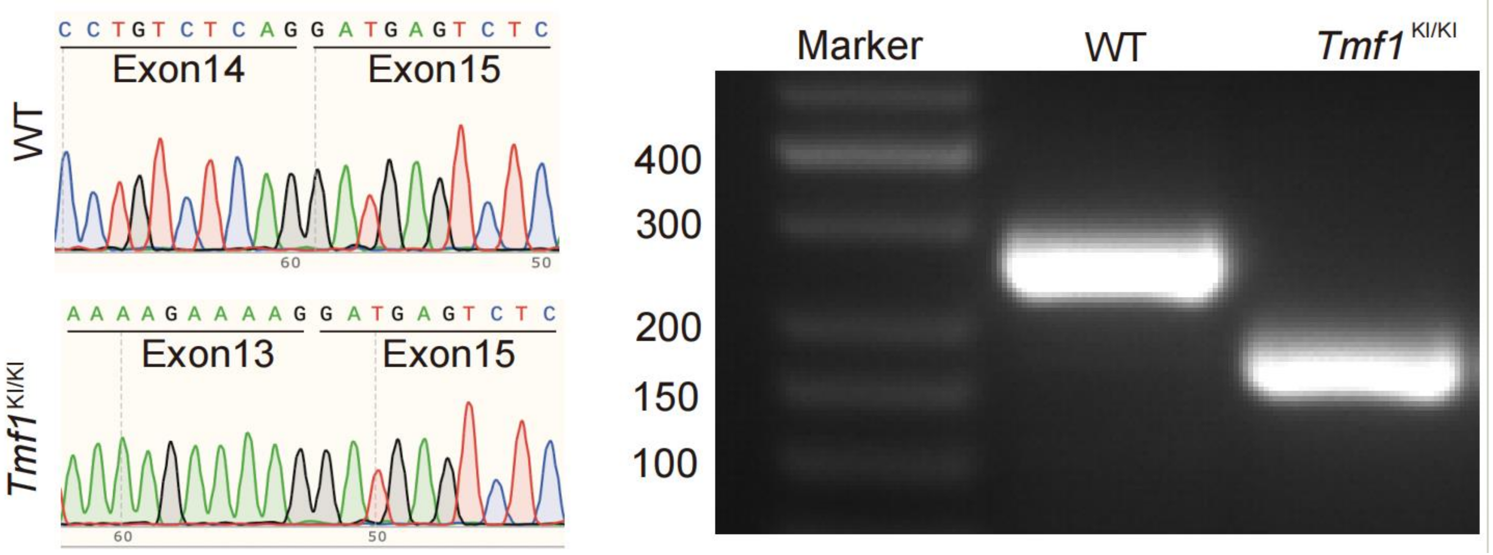

Figure S8. In vivo splicing function Verification of *Tmf1*$^{KI/KI}$ mice

Abnormal splicing of *TMF1* was verified by Sanger sequencing in vivo splicing function experiment using mRNA extracted from the testes of *Tmf1*$^{KI/KI}$ mice.

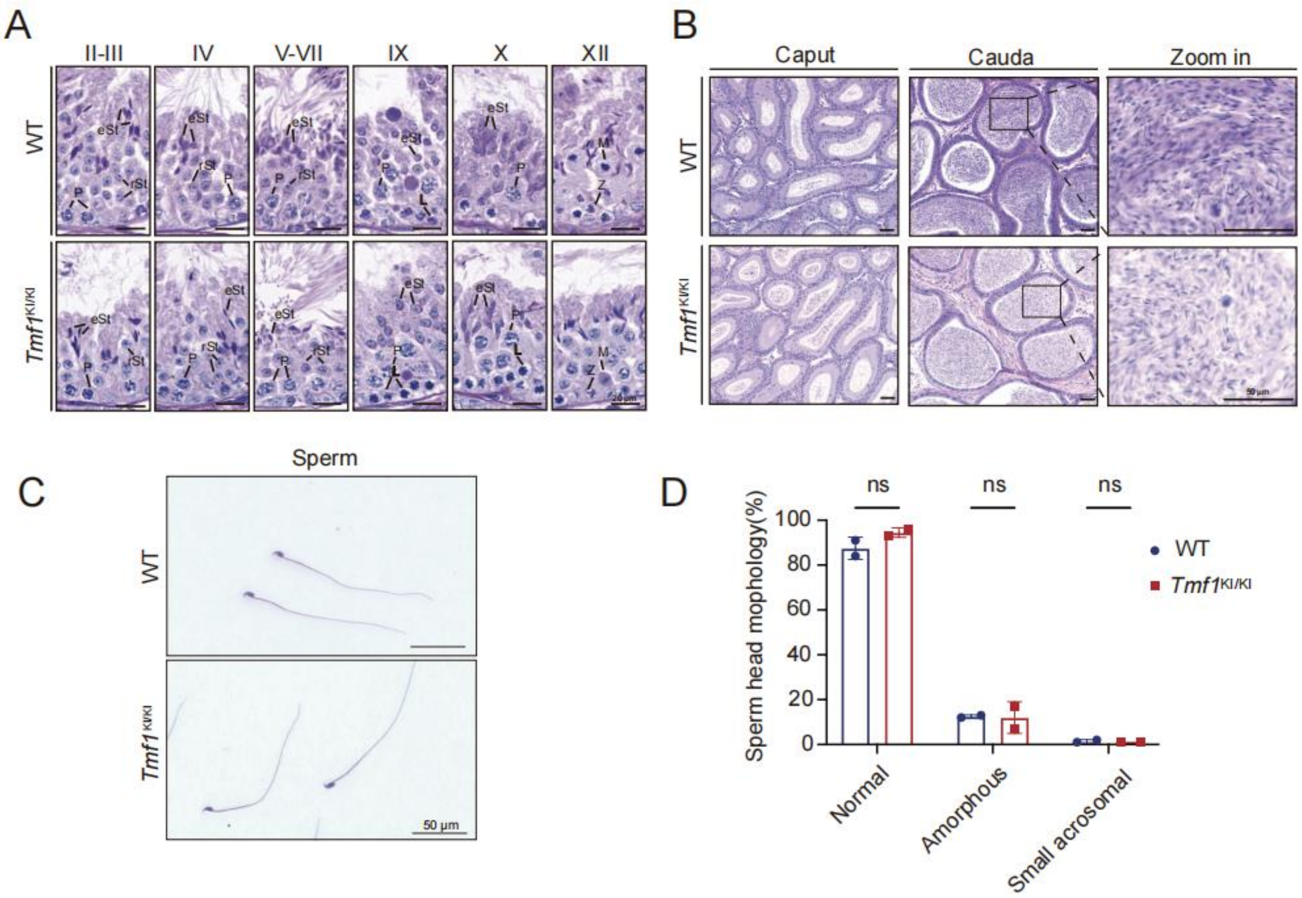

Figure S9. Morphological analysis of testes, epididymis sections and sperms in *Tmf1*$^{KI/KI}$ mice

A, PAS-hematoxylin staining of WT and *Tmf1*$^{KI/KI}$ mouse seminiferous tubules at different stages are shown. P pachytene, rST round spermatid, eST elongating/elongated spermatid, M, metaphase, Z, zygotene (Scale bars = 20μm). **B,** H&E staining of epididymis sections (Scale bars = 50 μm). **C-D,** Sperm morphological analysis in the WT and *Tmf1*$^{KI/KI}$ mouse under H&E staining (Scale bar = 50μm). One-way ANOVA, ns, not significance.
